# Enhancing Li‐S Battery Performance Through Low‐Concentration Electrolytes with Organic Se/Te Co‐Additives to Address Solubility and Kinetic Challenges

**DOI:** 10.1002/advs.75377

**Published:** 2026-04-24

**Authors:** Ruihua Li, Haiwei Wu, Hairu Wei, Wenhao Jia, Frederik Bettels, Leon Schenk, Zhihua Lin, Hanbin Liu, Guodong Liu, Zhijian Li, Lin Zhang

**Affiliations:** ^1^ College of Bioresources, Chemical and Materials Engineering Shaanxi University of Science & Technology Xi'an China; ^2^ Institute For Solid State Physics, Faculty of Mathematics and Physics Leibniz University Hannover Hannover Germany

**Keywords:** conversion kinetics, lithium–sulfur batteries, low‐concentration electrolyte, organic selenium/tellurium additives, solubility, solvation, synergistic catalysis

## Abstract

Lithium–sulfur (Li–S) batteries face low sulfur utilization and limited rate capability. Although electrolyte engineering is a key strategy for improving the conversion kinetics of lithium polysulfides (LiPS), achieving a balance between high energy density and high power density remains challenging. This study combines an ultra‐low concentration electrolyte with a dual‐functional hybrid organic selenium/organic tellurium additive (DPDSe/DPDTe). The system exhibits higher ionic conductivity and enhanced LiPS conversion kinetics. This is attributed to the high solubility of LiPS at low concentrations, which enhances the accessibility of active materials. By adjusting the mixing ratio of DPDSe/DPDTe, the formed DPDSe‐Te exhibits unique solvation structure regulation and synergistic catalytic effects. This Li–S battery delivers an initial specific capacity of 1103 mAh g^−1^ (65.9% sulfur utilization) at 0.5 C and retains 89.3% of its capacity after 100 cycles. Even at 2 C, a capacity of 783 mAh g^−1^ is achieved. The pouch cell exhibits a high energy density of 340 Wh kg^−1^ at 0.5 C with stable cycling. This low‐concentration, dual‐functional additive strategy synergistically addresses LiPS solubility limitations and kinetic bottlenecks, offering an effective route toward Li–S batteries with both high energy density and high‐power density.

## Graphical abstract

This work presents a low‐concentration electrolyte enabled by hybrid organic Se/Te additives (DPDSe/DPDTe) that restructures solvation, boosts polysulfide dissolution, and provides dual‐site synergistic catalysis, delivering high capacity and stable cycling in Li–S coin and pouch cells, supporting high‐energy, high‐power operation.

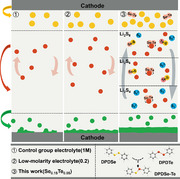

## 1 Introduction

Lithium–sulfur (Li–S) batteries have emerged as one of the most promising candidates for next‐generation energy storage systems, owing to their exceptionally high theoretical capacity (1675 mAh g^−1^) and energy density (2600 Wh kg^−1^), which surpass those of conventional lithium‐ion batteries [1, 2, 3]. Furthermore, sulfur, as the cathode active material, is naturally abundant, cost‐effective, and environmentally benign, further enhancing the commercial viability of Li–S batteries [4, 5, 6]. However, the practical application of conventional Li–S batteries is hindered by the lithium polysulfide (LiPS) shuttle effect and sluggish sulfur conversion kinetics, leading to low active material utilization and limited rate capability [7, 8, 9, 10, 11]. These challenges are further exacerbated under conditions of high sulfur loading and lean electrolyte, severely restricting their applicability in scenarios requiring high power density [12, 13].

Electrolyte engineering, particularly formulation optimization, is crucial for mitigating these issues [14, 15]. Conventional strategies often rely on highly concentrated electrolytes (HCEs) or localized highly concentrated electrolytes (LHCEs), which effectively suppress the dissolution of LiPS through strong solvation effects [16, 17]. However, inherent drawbacks such as high viscosity, poor wettability, and high cost limit their scalability [18]. In contrast, low‐concentration electrolytes (LCEs) offer advantages in terms of cost efficiency, low viscosity, reduced side reactions, and favorable interfacial kinetics, while also promoting sulfur dissolution to enhance the utilization of active material [19]. Nevertheless, solvent optimization alone is insufficient to fully resolve the intertwined challenges of active material utilization and reaction kinetics. Therefore, introducing functional additives to reconstruct interfacial reaction pathways has been proposed, ensuring stabilized electrochemical processes without compromising the inherent benefits of LCEs [20, 21].

Recent studies have highlighted the potential of organic selenium and tellurium compounds as functional additives in Li–S batteries [22, 23]. These materials exhibit unique redox activity and strong interactions with LiPS, enabling effective anchoring and catalytic conversion of soluble intermediates [24]. For instance, selenium‐doped polymer frameworks have demonstrated enhanced adsorption of short‐chain LiPS and accelerated redox conversions [25], while tellurium‐based mediators have been shown to improve the reaction kinetics of Li_2_S cathodes [26]. Despite these advancements, the application of mixed organic selenium/organic tellurium derivatives in LCEs solvation environments has not been fully explored. Moreover, the synergistic effects of these additives in modulating active material solubility and reaction kinetics within LCEs present a significant research gap.

To bridge this gap, this study proposes a dualstrategy electrolyte engineering approach. First, by precisely modulating the solvation structure, the concentration of the conventional lithium salt (LiTFSI) was reduced to 0.2 m to enhance cathode sulfur solubility and improve active material utilization. Second, and more critically, diphenyl diselenide (DPDSe) and diphenyl ditelluride (DPDTe) were concurrently introduced as novel synergistic additives for Li–S batteries under LCE conditions. The optimal combination concentration—0.15 m DPDSe and 0.05 m DPDTe—was systematically investigated. By virtue of the synergistic catalytic effect and interface regulation effect of DPDSe‐Te, this strategy enables the selective catalysis of the continuous solid–liquid–solid sulfur conversion reaction. It enhances LiPS adsorption and accelerates redox kinetics across all stages of polysulfide transformation, thereby overcoming kinetic limitations and enabling high sulfur utilization and high‐rate performance. The results demonstrate that with this novel strategy, the optimized low‐concentration hybrid electrolyte with DPDSe and DPDTe enables Li–S coin cells to deliver an ultra‐high initial specific capacity of 1103 mAh g^−1^ at 0.5 C, retaining a specific capacity of 985 mAh g^−1^ after 100 cycles, corresponding to a capacity retention rate of 89.3%. Moreover, the assembled Li–S pouch cell achieves an exceptional practical initial energy density of 340 Wh kg^−1^ at 0.5 C, thereby advancing the Li–S system toward practical application. This study not only provides a new strategy to resolve the conflict between low sulfur utilization and sluggish kinetics in Li–S batteries but also establishes a new paradigm for electrolyte design in multi‐electron transfer battery systems, propelling Li–S batteries toward practical high‐energy‐density storage applications.

## 2 Experimental Section

### 2.1 Raw Materials

Sublimated sulfur (S_8_,  > 99.9%) and multi walled carbon nanotubes (CNT, > 98%) were purchased from Zhongke Jinyan Technology Co., Ltd. (Beijing, China). Polyacrylonitrile (LA132, 15%) was purchased from Tianjin Annuohe New Energy Technology Co., Ltd. (Tianjin, China). Lithium sulfide (Li_2_S, > 99.9%), lithium bis(trifluoromethanesulphonyl)imide (LiTFSI, > 99.9%), and diphenyl ditelluride (DPDTe, > 98%) were purchased from Shanghai Macklin Biochemical Co., Ltd. (Shanghai, China). Dimethyl ether of ethylene glycol (DME, > 99.9%) was purchased from Titan Technology Co., Ltd. (Shanghai, China). Diphenyl diselenide (DPDSe, > 96%), 1,3‐dioxolane (DOL, > 99.9%), and lithium nitrate (LiNO_3_, > 99.9%) were purchased from Aladdin Reagent Co., Ltd. (Shanghai, China). PP diaphragm (Celgard 2500, 25 µm) was purchased from Bolton Diaphragm Products Co., Ltd. (Shanghai, China).

### 2.2 Synthesis of Cathode Materials

Sublimated sulfur and carbon nanotubes were ground in a mortar at a weight ratio of 2:1 for 30 min to ensure uniform mixing. The resulting mixture was then melted at 155°C in an argon atmosphere for 12 h to obtain the powder material. Subsequently, it was stirred with the binder LA132 in deionized water at a ratio of 9:1 for 30 min until completely dissolved. Then, it was evenly coated on the carbon‐coated aluminum foil using a 400 µm coater. The electrode with a sulfur loading of approximately 2.3 mg cm^−2^ (S: 60 wt.%) obtained by the coating method was the cathode used in this study. In addition, a high‐loading electrode of approximately 4.4 mg cm^−2^ (S: 60 wt.%) was prepared by the coating method using a 500 µm coater.

The carbon nanotubes and LA132 were ground and mixed evenly in a weight ratio of 9:1. The sulfur‐free carbon paper used was prepared on the carbon‐coated aluminum foil using the same coating method as mentioned above.

### 2.3 Preparation of Electrolytes

A commercial electrolyte (1 m LiTFSI with 3 wt.% LiNO_3_ in DOL/DME, v:v = 1:1) was used as the control group.

Under an argon atmosphere, a low‐concentration electrolyte (denoted as 0.2) was prepared by adding 0.115 g (0.2 m) of LiTFSI and 0.0414 g (3 wt.%) of LiNO_3_ into a glass vial, followed by dilution with a DOL/DME mixture (v:v = 1:1) to a total volume of 2 mL. Based on the above formulation, comparative electrolyte samples were prepared by further adding: 0.1248 g (0.2 m) of DPDSe (denoted as 0.2Se), 0.1638 g (0.2 m) of DPDTe (denoted as 0.2Te), and a mixture of 0.234 g (0.15 m) DPDSe + 0.1024 g (0.05 m) DPDTe (denoted as Se_0.15_Te_0.05_). The configured electrolytes were allowed to stand in a glove box for 12 h.

### 2.4 Preparation of Lithium Polysulfide Solutions

Saturated Li_2_S_4_ Solutions in the Five Electrolytes: Under an argon atmosphere, 0.161 g of lithium sulfide (Li_2_S) and 0.337 g of sulfur powder were added to 2 mL of each of the five electrolytes at a molar ratio of 1:3. The bottles containing the mixtures were stirred in an oil bath at 80°C for 48 h until the solid powders were completely saturated and the solutions turned brownish‐red. After cooling, a noticeable precipitate of sulfur was observed at the bottom of the bottles.

Saturated Li_2_S_6_ Solutions in the Five Electrolytes: Using the same method described above, 0.138 g of Li_2_S and 0.481 g of sulfur were dissolved in 2 mL of each electrolyte at a molar ratio of 1:5 to prepare saturated Li_2_S_6_ solutions.

Saturated Li_2_S_8_ Solutions in the Five Electrolytes: Using the same method described above, 0.115 g of Li_2_S and 0.561 g of sulfur were dissolved in 2 mL of each electrolyte at a molar ratio of 1:7 to obtain saturated Li_2_S_8_ solutions.

0.5 m Li_2_S_8_ Solutions in the Five Electrolytes: Under an argon atmosphere, 0.046 g of Li_2_S and 0.225 g of sulfur powder were mixed into 2 mL of each electrolyte at a molar ratio of 1:7. The bottles were stirred in an oil bath at 80°C for 48 h until complete dissolution of the solids was achieved, resulting in a brownish‐red solution after cooling down.

### 2.5 Material Characterization

The surface morphology of the electrode was examined using a scanning electron microscope (SEM) (Regulus 8100). The components and interactions within the prepared electrolyte were characterized by Raman spectroscopy (THEM/DXRxi). UV–vis absorption spectra were measured and identified by a UV–vis spectrophotometer (Lambda25). The wettability of the interface between the electrolyte and the electrode was observed by using a contact angle tester (OCA20). The conductivity and resistivity of the electrolyte were analyzed by using a conductivity meter (DDSJ‐307F). ^1^H nuclear magnetic resonance (NMR) characterization was performed using a nuclear magnetic resonance spectrometer, with deuterated chloroform (CDCl_3_) as the solvent. All sample preparation, transferring, and testing were conducted under an argon atmosphere.

### 2.6 Electrochemical Measurements

CR2016 coin‐type Li–S batteries were fabricated by assembling self‐made coated cathodes, lithium metal anodes, commercial polypropylene (PP) separators, and different electrolytes. In a common battery, the sulfur loading on the cathode is controlled at approximately 2.3 mg cm^−2^, and about 20 µL of electrolyte is added. The ratio of electrolyte to sulfur (E/S) is about 7.7 µL mg^−1^. 10 µL of electrolyte was added onto the Li metal anode. A total of 30 µL of electrolyte was added to one battery. In the high‐loading battery, the sulfur loading on the cathode was controlled at approximately 4.4 mg cm^−2^, and the E/S ratio was controlled at 4.1 µL mg^−1^. The batteries were subjected to galvanostatic charge–discharge (GCD) tests, constant current intermittent titration (GITT) tests, and lithium sulfide deposition tests using battery testers (NEWARE CT 4008Tn‐5V10mA‐164 and LAND CT 2001 A). The cyclic voltammetry (CV) and electrochemical impedance spectroscopy (EIS) of Li–S cells and symmetric cells were measured using an electrochemical workstation (CHI 760 E). All electrochemical tests of the battery were conducted at room temperature.

Further experimental details are described in the supporting information.

## 3 Result and Discussion

**FIGURE 1:**
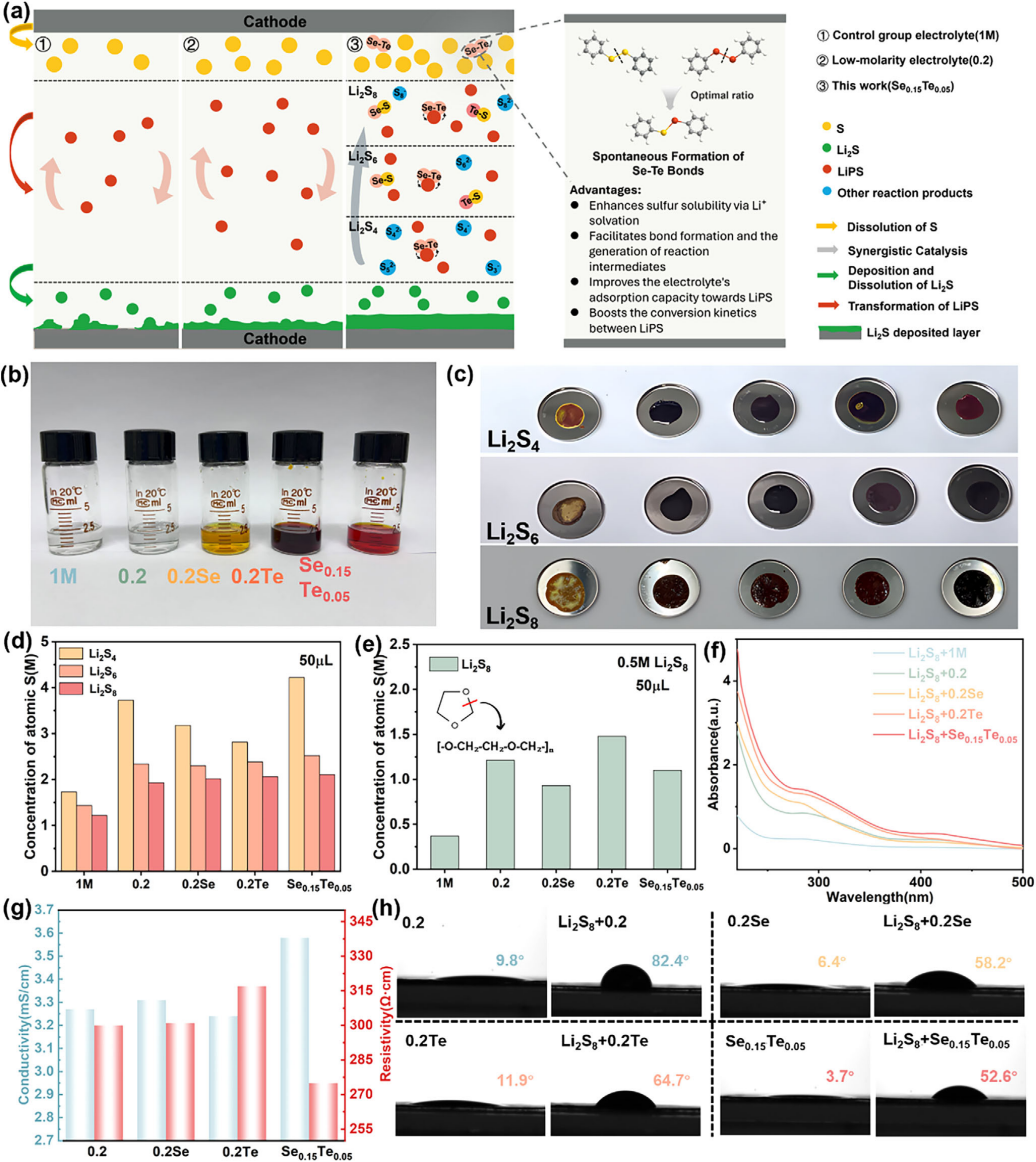
(a) Mechanism diagram for achieving high sulfur solubility and synergistic catalysis. (b) Photographs of the five electrolytes: 1M, 0.2, 0.2Se, 0.2Te, and Se_0.15_Te_0.05_. (c) Photographs and (d) corresponding solubility measurements of different polysulfides from the five electrolytes after solvent evaporation in coin‐cell cases. (e) Verification of the influence of DOL ring‐opening polymerization on solubility. (f) UV–vis spectra of Li_2_S_8_ in different electrolytes. (g) Ionic conductivity of 0.2, 0.2Se, 0.2Te, Se_0.15_Te_0.05,_ and (h) Contact angle photos before and after adding Li_2_S_8_.

In Li–S batteries, the electrolyte system composed of LiTFSI and lithium nitrate (LiNO_3_) dissolved in a 1,3‐dioxolane/dimethoxyethane (DOL/DME) mixed solvent has become the mainstream research choice [27]. According to numerous research reports, the dissolution of sulfur in electrolytes is a double‐edged sword [28]. In the current mainstream Li‐S battery systems with high sulfur loading and lean electrolyte, promoting the rapid conversion of sulfur is regarded as one of the feasible strategies to improve sulfur utilization, due to the limitations of electron conduction bottlenecks and polysulfide diffusion [29]. Therefore, how to increase the maximum solubility of sulfur and its discharge products while achieving their rapid catalytic conversion in the liquid phase has become a critical issue urgently needing to be addressed in this field. Against this backdrop, this study is based on the traditional 1  LiTFSI electrolyte system, regulating the solvation structure of the electrolyte through the dual approaches of “reducing solute concentration” and “introducing catalysts” to ultimately achieve rapid sulfur conversion. It is noteworthy that the catalytic solutes (DPDSe and DPDTe) introduced in this study are a class of substances that can simultaneously solvate lithium ions and interact with sulfur anions. As shown in Figure 1a, Se and Te can coordinate with Li^+^ to reconstruct the solvation environment, thereby promoting the dissolution of polysulfides. Meanwhile, the spontaneously formed Se‐Te bonds provide abundant dual active sites for anchoring polysulfides and catalyzing their rapid conversion, which effectively resolves the trade‐off between high solubility and fast kinetics.

The following sections will elaborate on their dual catalytic mechanism in low‐concentration electrolyte systems. Finally, a low‐concentration electrolyte system (0.2 m LiTFSI with 3 wt.% LiNO_3_ in DOL/DME = 1:1 (v:v)) was adopted, and the combined concentrations of DPDSe and DPDTe were systematically investigated. For comparison with the commercial electrolyte (1 m LiTFSI with 3 wt.% LiNO_3_ in DOL/DME = 1:1 (v:v)) (1M), a blank solution (0.2 m LiTFSI with 3 wt.% LiNO_3_) (0.2) was prepared. Based on the above findings, a preliminary experiment (Figure S1) was performed, and three modified electrolytes were further prepared for comparative analysis: 0.2 m DPDSe (denoted as 0.2Se), 0.2 m DPDTe (denoted as 0.2Te), and a mixture of 0.15 m DPDSe and 0.05 m DPDTe (denoted as Se_0.15_Te_0.05_). As shown in Figure 1b, both the 1M and 0.2 solutions appeared transparent. After adding DPDSe and DPDTe, the solutions turned yellow and brown, respectively, while Se_0.15_Te_0.05_ exhibited an intermediate red color. All solutions remained stable over time without phase separation or precipitation.

To evaluate the solubility of LiPS in different electrolytes, saturated Li_2_S_4_, Li_2_S_6_, and Li_2_S_8_ solutions were prepared in each of the five electrolytes. Take 50 µL of different solutions, place them in coin‐cell cases, and heat at 45°C in a glovebox for 48 h to evaporate the solvent. After cooling, the residues were observed. As shown in Figure 1c, the 0.2, 0.2Se, 0.2Te, and Se_0.15_Te_0.05_ samples displayed darker brown colors compared to the pale‐yellow residue of the 1M sample, indicating higher LiPS precipitation. After subtraction of tare mass (Net residual mass of the polysulfide‐containing electrolyte = total mass − mass of the electrode casing − mass of the inherent nonvolatile solutes in the electrolyte (LiTFSI, LiNO_3_, DPDSe, and DPDTe)), the solubility results are shown in Figure 1d. It can be observed that the solubility of LiPS increases significantly with the reduction of electrolyte concentration. This phenomenon is attributed to the increased number of free solvent molecules at low concentrations, which enhances the solvating ability of polysulfides. Furthermore, among all saturated solutions of Li_2_S_4_, Li_2_S_6,_ and Li_2_S_8_, the Se_0.15_Te_0.05_ electrolyte exhibits the highest solubility. The two introduced substances (DPDSe and DPDTe) are likely to exert a solvating effect on lithium ions, thereby maintaining high solubility while incorporating new solutes. 0.2Se and 0.2Te are relatively stable for long‐chain Li_2_S_8_ and Li_2_S_6_, but the solubility of Li_2_S_4_ shows a downward trend, with 0.2Te being more pronounced. This focuses on the larger atomic radius and higher polarizability of Te compared to Se. The resulting stronger intermolecular interactions and increased steric hindrance of DPDTe significantly increase the viscosity of the electrolyte system when added in excess. This inevitably reduces the fraction of free solvent molecules available for Li^+^ solvation, thereby suppressing the solubility of the short‐chain Li_2_S_4_. The electrolyte Se_0.15_Te_0.05_ exhibits the highest solubility in all saturated solutions. Especially Li_2_S_4_, this indicates that by precisely regulating the addition ratio of additives, the solvation effect of LiPS can be effectively controlled, promoting a balance between maintaining the excellent solvation effect of LiPS and the increase in system viscosity caused by excessive addition. This might be due to the exchange effect brought about by the SeTe mixture, which induced a solvation effect of 1+1 > 2. For detailed analysis, please refer to the following experimental section.

On the other hand, to verify the influence of ring‐opening polymerization of DOL solvent during the evaporation process on the solubility results, the following verification experiment was conducted [30]. Five 0.5 m Li_2_S_8_ solutions of electrolyte were prepared. A fixed volume of 50 µL was taken from each solution and placed into an electrode case. After the solvent was completely evaporated to dryness, the sample was weighed with the tare deducted. The obtained masses are shown in Figure 1e. Except for 1 Mm of high‐concentration lithium salt, the mass of Se_0.15_Te_0.05_ was only slightly greater than 0.2Se. This may be attributed to Se and Te atoms have a stronger electron‐donating ability and a larger atomic radius than the oxygen atoms in DOL molecules, which can more effectively stabilize the Lewis acids (mainly Li^+^) that initiate polymerization reactions, significantly inhibiting or terminating the initiation step of DOL ring‐opening polymerization. A large part of the Li^+^ originates from LiPS, which also proves the solvating effect of Se and Te on LiPS. The reliability of the results in Figure 1d was verified once again.

To further verify the solvation effect brought by the additive, five electrolytes of Li_2_S_8_ were further characterized by Raman spectroscopy. As shown in Figure S2, Raman spectroscopy indicates that all five solutions exhibit several characteristic peaks at 393, 447, and 495 cm^−1^, demonstrating the successful preparation of Li_2_S_8_ in the five electrolytes. Five solutions of Li_2_S_8_ were diluted to 2 mm and tested by the ultraviolet spectrophotometer. The results are shown in Figure 1f. The peak intensity of the diluted Se_0.15_Te_0.05_ + Li_2_S_8_ solution at 280 nm was significantly higher than that of the other four solutions. These results indicate that on a low concentration basis, the addition of Se_0.15_Te_0.05_ further promoted the dissolution of S and may significantly improve the utilization rate of the sulfur when using in Li–S batteries. Furthermore, as shown in Figure S3, the ionic conductivity of the 1M electrolyte was 11.82 mS cm^−1^, which is considerably higher than that of the low‐salt‐concentration systems (0.2 m) in Figure 1g due to its higher lithium salt concentration. Nevertheless, the Se_0.15_Te_0.05_ electrolyte exhibited an ionic conductivity of 3.58 mS cm^−1^ and a resistivity of 275 Ω·cm, outperforming the 0.2, 0.2Se, and 0.2Te samples. This improved conductivity may be attributed to the introduced DPDSe and DPDTe, reducing the chelation around Li^+^ ions, thereby facilitating their migration within the electrolyte [31]. To more intuitively observe the influence of solubility changes on wettability, Figure 1h shows the contact angles of the four electrolytes with the electrode sheet in a low lithium salt concentration system (0.2 m). Compared with 0.2, the contact angles of 0.2Se and Se_0.15_Te_0.05_ are further reduced to varying degrees. Meanwhile, excessive addition of DPDTe to 0.2Te will have the opposite effect, increasing the contact angle. When Li_2_S_8_ was introduced, the Se_0.15_Te_0.05_ electrolyte with the highest solubility (consistent with Figure 1d) exhibited the smallest contact angle. This once again confirms that the appropriate proportion of DPDSe and DPDTe incorporation can comprehensively cover the electrode surface and pores, significantly increase the effective mass transfer interface area between the electrode and the electrolyte, and enhance the mass transfer rate.

**FIGURE 2:**
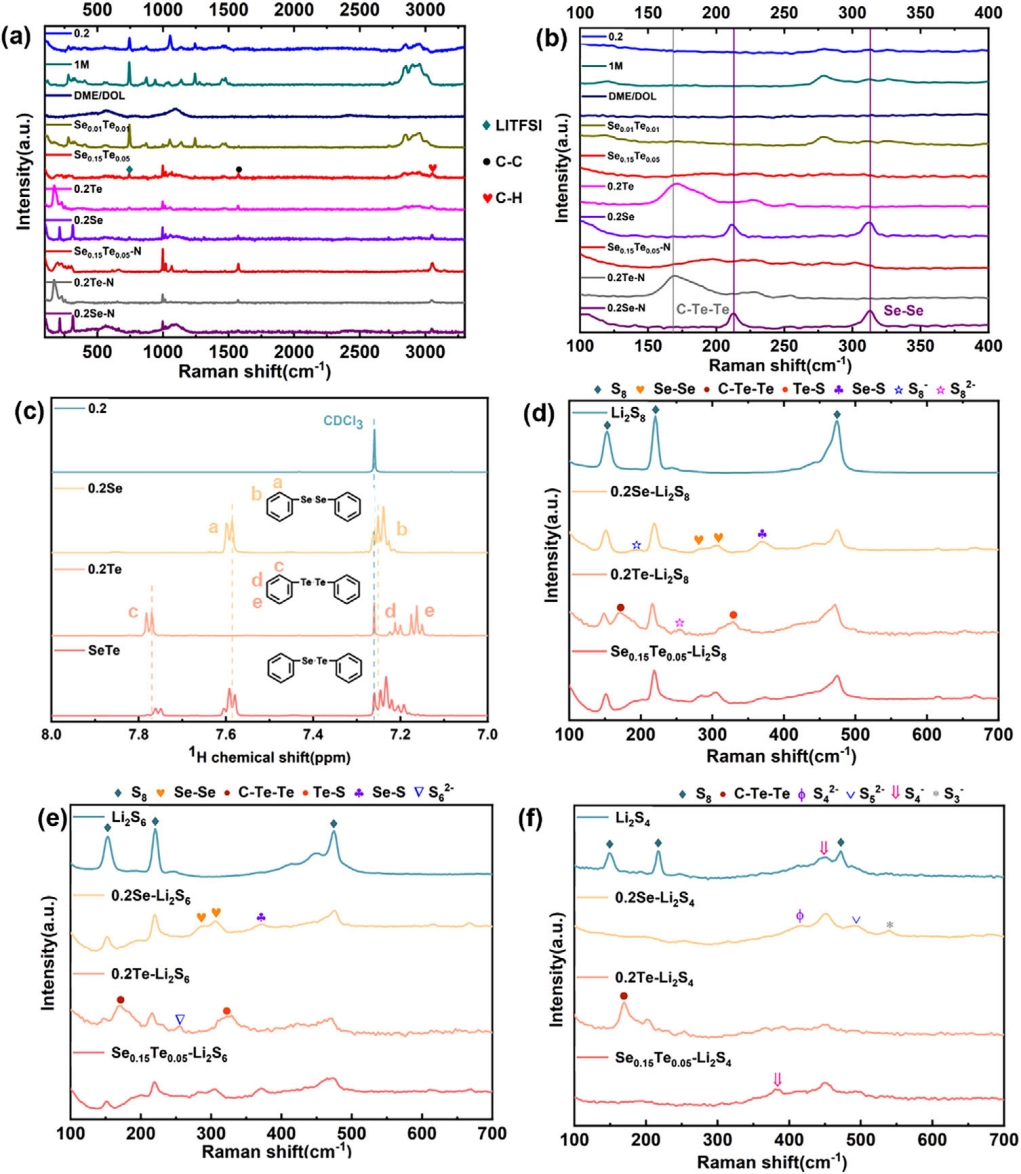
Raman spectra of (a) different electrolytes (100–3500 cm^−1^) and (b) (100–400 cm^−1^). (c) ^1^H NMR spectra of different electrolytes. Raman spectra of (d) Li_2_S_8_, (e) Li_2_S_6_, and (f) Li_2_S_4_ upon addition to 0.2Se, 0.2Te, and Se_0.15_Te_0.05_ electrolytes.

To study the differences in solvation environments among various electrolytes, other control electrolytes (1 m LiTFSI with 2 wt.% LiNO_3_, 0.01 m DPDSe, 0.01 m DPDTe in DOL/DME = 1:1(v:v)) (Se_0.01_Te_0.01_) were also prepared [32]. And lithium salt‐free electrolytes—including (0.2 m DPDSe in DOL/DME = 1:1(v:v)) (0.2Se–N), (0.2 Mm DPDTe in DOL/DME = 1:1(v:v)) (0.2Te‐N), and (0.15 m DPDSe + 0.05 m DPDTe in DOL/DME = 1:1(v:v))(Se_0.15_Te_0.05_‐N) were also prepared and analyzed using Raman spectroscopy. As shown in Figure 2a, the characteristic peak observed at 742 cm^−1^ is attributed to the coordination between Li^+^ and TFSI^−^ [33, 34]. The peak at 1570 cm^−1^ corresponds to the C‐C bond vibrations in DPDSe and DPDTe, confirming the successful preparation of the various electrolytes. Additionally, the C‐H stretching vibration of unsaturated carbon atoms at 3052 cm^−1^ further supports this conclusion. Figure 2b more intuitively illustrates the influence of DPDSe and DPDTe on the solvation structure within the electrolyte. A comparison between the spectra of 0.2Se and 0.2Se‐N reveals a red shift in the characteristic Se‐Se peaks located at 213 and 313 cm^−1^. This shift indicates that the highly electronegative Se atoms in DPDSe preferentially coordinate with Li^+^ ions. This coordination causes the electron cloud of the Se‐Se bond to shift toward the Li^+^ ion, weakening the bond strength and facilitating the formation of contact ion pairs (CIP) [35]. In contrast, a comparison between 0.2Te and 0.2Te‐N shows a blue shift in the C‐Te‐Te characteristic band at 172 cm^−1^. This suggests that the large atomic radius of Te in DPDTe requires more spatial accommodation during coordination with Li^+^. The strong coordination distorts the C‐Te‐Te bond angle and restricts its vibrational freedom, leading to spatial compression and increased bond rigidity, thereby promoting the formation of anion‐cation aggregates (AGG) [36]. Notably, in the case of Se_0.15_Te_0.05_ and Se_0.15_Te_0.05_‐N, the characteristic Raman signals for both Se‐Se and C‐Te‐Te bonds disappear. This observation suggests the occurrence of dynamic ligand exchange and spontaneous bonding between DPDSe and DPDTe in the electrolyte. The Raman signal for a potential Se‐Te bond is inherently weak. The formation was further confirmed by the ^1^H NMR spectroscopic results. As shown in Figure 2c, Se_0.15_Te_0.05_ exhibits a more complex spectral feature, containing some peak characteristics of both DPDSe and DPDTe. Neither the positions nor the shapes of these peaks correspond to those of pure DPDSe or pure DPDTe anymore; in addition, peak broadening and desymmetrization phenomena are observed [37]. Its underlying mechanism involves a spontaneous chalcogen exchange reaction driven by the homolysis of relatively weak Se‐Se and Te‐Te bonds. This process generates transient phenyl chalcogen radicals (Ph‐Se• and Ph‐Te•), which then undergo more favorable heteronuclear recombination to form asymmetric Ph‐Se‐Te‐Ph species (DPDSe‐Te), thereby establishing a dynamic equilibrium [38]. These observations indicate that the original Se‐Se and Te‐Te bonds have broken, and new Se‐Te bonds have formed in the structure.

To gain deeper insight into the mechanistic role of additives in the polysulfide conversion process, this study systematically compared the Raman spectra of various polysulfides (Li_2_S_8_, Li_2_S_6_, Li_2_S_4_) in different electrolyte systems (Figure 2d–f). As shown in Figure 2d, compared to the characteristic peaks of S_8_ at 156, 223, and 476 cm^−1^ in the pure Li_2_S_8_ electrolyte [39], a peak attributed to S_8_^−^ appears at 187 cm^−1^ in the 0.2Se–Li_2_S_8_ electrolyte, and a characteristic band of Se‐S bond is detected at 369 cm^−1^ [34, 40]. Furthermore, the originally weak signal of the Se‐Se bond at 286 cm^−^^1^ (referenced from Figure 2b) becomes pronounced, indicating the participation of a significant portion of DPDSe in the reaction [41]. Concurrently, in the 0.2Te‐Li_2_S_8_ electrolyte, a characteristic feature of S_8_^2−^ is observed at 253 cm^−1^, accompanied by a vibrational peak of the Te‐S bond at 329 cm^−1^ [32]. These marked spectral changes confirm that both Se and Te additives effectively catalyze the conversion of Li_2_S_8_. After the two additives were mixed, the Se_0.15_Te_0.05_‐Li_2_S_8_ electrolyte retained various characteristic signals catalyzed by 0.2Se‐Li_2_S_8_ and 0.2Te‐Li_2_S_8_. As shown in the results in Figure 2e, in the Li_2_S_6_ system, the effect of the 0.2Se additive is reflected by the emergence of the characteristic band of the Se‐S bond [40]. In contrast, the 0.2Te additive not only induces the formation of a Te‐S bond but also gives rise to a characteristic feature of S_6_^2−^ at 255 cm^−1^, indicating its influence on the Li_2_S_6_ conversion pathway [34]. Se_0.15_Te_0.05_‐Li_2_S_6_ also retains the catalytic results of both. The smaller peak intensity of S_6_^2−^ might be due to the relatively small proportion of DPDTe in the mixed system. For the Li_2_S_4_ system in Figure 2f, pure Li_2_S_4_ exhibits a distinct characteristic peak of S_4_^−^ at 451 cm^−1^. Upon introduction of the 0.2Se additive (forming 0.2Se‐Li_2_S_4_), additional characteristic peaks are observed alongside the S_4_^−^ peak, which are attributed to S_4_^2−^ (415 cm^−1^), S_5_^2−^ (494 cm^−1^), and S_3_^−^ (539 cm^−1^). In the Se_0.15_Te_0.05_ mixed additive system, an additional characteristic S_4_^−^ signal emerged at 382 cm^−1^ while retaining the 0.2Se‐Li_2_S_4_ characteristic peak, which was attributed to the synergistic effect of Se/Te [34]. Notably, in all three polysulfide systems, a pronounced characteristic band of the C‐Te‐Te bond was observed for the 0.2Te electrolyte [36], indicating an excess presence of the DPDTe additive (at 0.2 m) across different conversion stages. This discovery verifies that the proportion of DPDTe should be reduced in the two‐additive mixed system. To exclude the possibility of spontaneous reactions between the additives and elemental sulfur, a control experiment was also conducted by adding sulfur powder to the Se_0.15_Te_0.05_ electrolyte (denoted as Se_0.15_Te_0.05_‐S), followed by prolonged stirring and subsequent Raman testing. As shown in Figure S4, aside from the characteristic peaks of S_8_, no significant new peaks were detected. This result definitively rules out any spontaneous reaction between the additives and sulfur in the absence of an electrochemical process, further confirming that the catalytic effect of the additives on polysulfide conversion occurs specifically under electrochemically driven conditions. In summary, by accurately adjusting the proportion to mix the DPDSe and DPDTe additives, the surplus of some additives that did not participate in the reaction was avoided. This not only retained the catalytic effect of individual additives but also promoted the formation of new chemical bonds and induced the generation of intermediate substances through the interaction of dual active sites with polysulfides at different stages. This interaction significantly enhances the adsorption capacity of the electrolyte for polysulfides, improves the utilization rate of active substances, and effectively promotes the transformation kinetics among different polysulfides.

**FIGURE 3:**
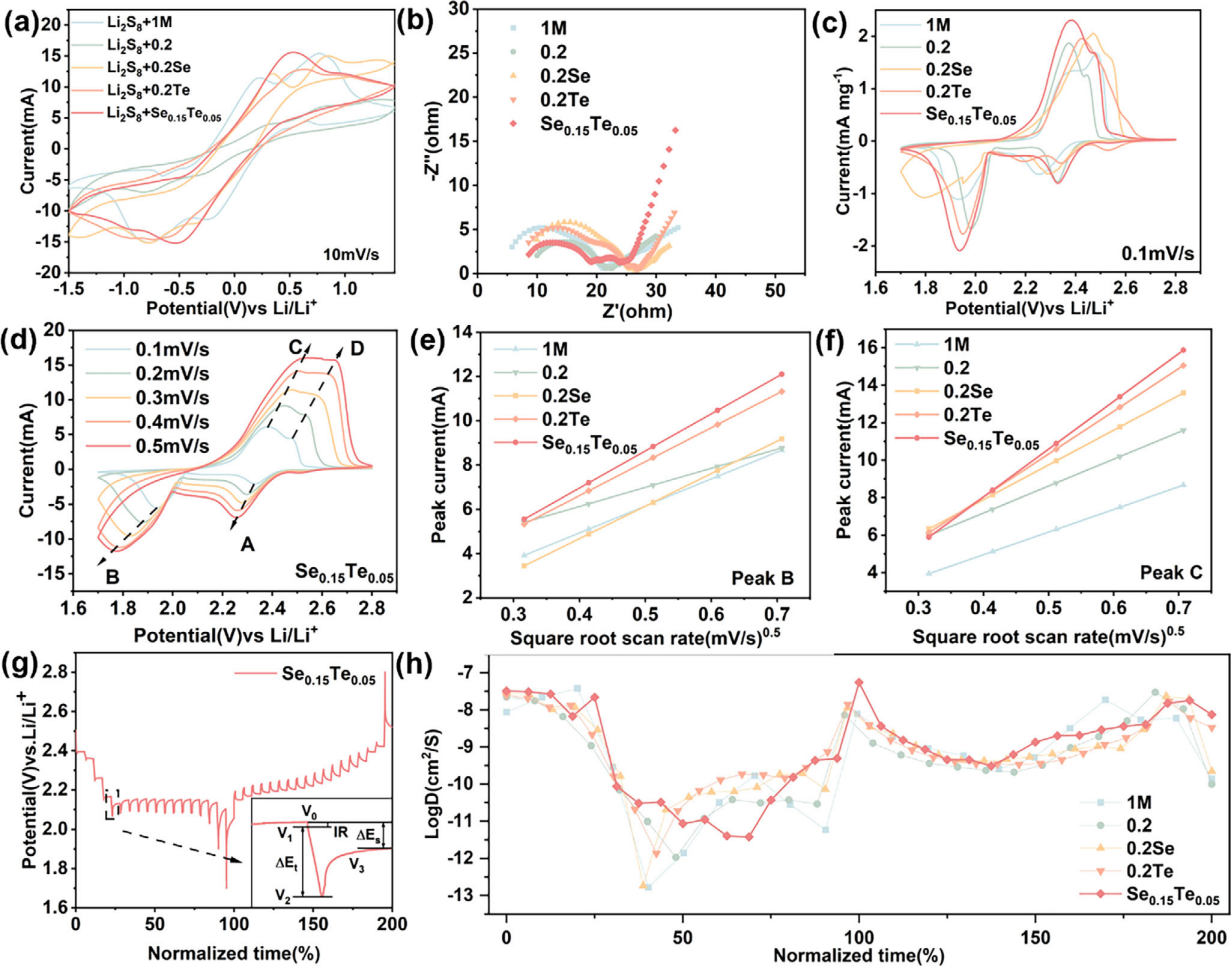
(a) CV curves at 10 mV s^−1^ for symmetric cells containing Li_2_S_8_ in 1M, 0.2, 0.2Se, 0.2Te, and Se_0.15_Te_0.05_ electrolytes. (b) EIS spectra and (c) CV curves at 0.1 mV s^−1^ for batteries using the five electrolytes. (d) CV curves of the Se_0.15_Te_0.05_ battery at scan rates from 0.1 to 0.5 mV s^−1^. CV peak current of (e) peak B and (f) peak C versus the square root of the scan rate. (g) GITT curve and its local magnification for the Se_0.15_Te_0.05_ battery. (h) Lithium‐ion diffusion coefficients for batteries using the five electrolytes.

In Li–S battery research, the relatively low solubility of LiPS and low conversion kinetics in the electrolyte restrict the utilization rate of S_8_ [42]. To further investigate the diffusion and conversion kinetics facilitated by DPDSe and DPDTe additives in Li–S batteries, high‐loading S cathode materials were fabricated in this study. The SEM image in Figure S5 shows that S in the electrode powder is uniformly combined with the conductive agent (CNT). As illustrated in Figure S6, the as‐prepared electrode exhibits a homogeneous distribution of sublimed sulfur and CNT across the current collector surface. The resulting porous structure after vacuum heating provides abundant ion transport channels. Symmetric cells were assembled using 0.5 m Li_2_S_8_ solutions (in the five electrolytes) as the electrolytes. Cyclic voltammetry (CV) tests for these symmetric cells were performed within a voltage range of ‐1.5 to 1.5 V at scan rates of 5 and 10 mV s^−1^, as shown in Figure 3a and Figure S7. Compared to the initial two pairs of redox peaks observed in the 1M cell, the CV curve of the Se_0.15_Te_0.05_ cell exhibits a merged pair of peaks. This merging is attributed to reactions between Li_2_S_8_ and DPDSe/DPDTe. When comparing the capacitive current responses under low‐concentration conditions, cells with 0.2Se and 0.2Te electrolytes demonstrate significantly higher peak currents than the 0.2 cell, indicating that both DPDSe and DPDTe enhance the redox kinetics of LiPS. Notably, the Se_0.15_Te_0.05_ symmetric cell exhibits a higher current response than all other cells. Furthermore, the Se_0.15_Te_0.05_ cell shows the narrowest peak separation, confirming its superior effectiveness in enhancing the lithiation/delithiation kinetics of polysulfide conversion [43]. In contrast, the smaller current response of the 0.2 cell and the wider peak separations observed in the 1M, 0.2Se, and 0.2Te cells are due to their inferior conductivity and sluggish reaction kinetics.

Subsequently, electrochemical impedance spectroscopy (EIS) tests were conducted on the Li–S battery, as shown in Figure 3b. Compared with other batteries, the Se_0.15_Te_0.05_ battery has a smaller semicircle diameter (related to the charge transfer resistance R_ct_), which indicates that the introduction of DPDSe and DPDTe is more effective in enhancing the charge transfer of LiPS transformation. In addition to 1M, the intercept in the high‐frequency region (related to the solution resistance R_s_) also proves that Se_0.15_Te_0.05_ has a smaller solution resistance value. The slope in the low‐frequency region (related to Warburg impedance Z_w_) characterizes the diffusion impedance of Li^+^ in the electrode material. It is observed that Se_0.15_Te_0.05_ exhibits a steeper slope, which indicates a smaller Z_w_ and a faster Li^+^ diffusion rate. CV tests were also conducted on the Li–S battery to further confirm the advantages of DPDSe, DPDTe, and their appropriate mixtures in promoting kinetics at low concentrations. As shown in Figure 3c, d, and Figure S8, during the discharge process, the two peaks A and B correspond to the reduction of S_8_ to soluble LiPS and LiPS to solid Li_2_S_2_/Li_2_S. During the charging process, the oxidation peaks of C and D respectively represent the reverse transformation of Li_2_S_2_/Li_2_S oxidizing the long chain LiPS and LiPS oxidizing to S_8_ [44, 45]. Both the reduction peak and the oxidation peak of Se_0.15_Te_0.05_ exhibit the strongest current response. It indicates that Se_0.15_Te_0.05_ can effectively promote the rapid reversible transformation among sulfur, LiPS, and Li_2_S_2_/Li_2_S in Li–S batteries. Furthermore, during the process of S_8_ dissolving into LiPS, the 0.2Se battery exhibited a slightly higher current response than the 0.2Te battery. In contrast, the presence of 0.2Te significantly enhanced the conversion from LiPS to Li_2_S_2_/Li_2_S, as demonstrated by the significantly higher current response at peaks A and B, while Se_0.15_Te_0.05_ consistently maintained a higher current response than other batteries. These different dynamic current variations concisely demonstrate the synergistic catalytic effect of the DPDSe‐Te catalyst formed after mixing during the charging and discharging process. Furthermore, as the scanning rate increases, Se_0.15_Te_0.05_ exhibits the highest peak current, which also confirms its rapid polysulfide conversion kinetics.

On the other hand, in combination with the Randles–Sevcik Equation (1)

(1) $$I_{p}=\left(2.69\times 10^{5}\right)\;n^{1.5}SD^{0.5}\Delta C_{Li}V^{0.5}$$

Here, I_p_ represents the peak current, n is the number of electrons, S is the geometric area of the cathode, D is the diffusion coefficient of lithium ions, v is the scanning rate, and ΔC_Li_ is the change in Li^+^ concentration during the electrochemical reaction process. The peak values of the oxidation and reduction processes are selected to determine the diffusion coefficient. The slope of the curve (Ip/v^0.5^) is positively correlated with the diffusion coefficient of lithium ions. The greater the slope of the curve, the higher the diffusion coefficient of lithium ions [46, 47]. As shown in Figure 3e, f, and Figure S9, compared with the other four batteries, the Se_0.15_Te_0.05_ battery shows a greater slope, which means that the diffusion rate of lithium ions is faster.

DPDSe‐Te significantly promotes the lithiation process of Li^+^ and the transfer of Li^+^ in electrochemical reactions. Reaction and diffusion kinetics can also be characterized by constant current intermittent titration (GITT) technology [48]. The GITT test is a pulse‐relaxation cycle. A pulse refers to a short period of time when a small current passes through, and relaxation refers to a long period of time when no current passes through. To fully stabilize the relaxation voltage, an intermittent charge–discharge strategy of constant current for 1 h and rest for 5 h was adopted, and the test was conducted at a small current of 0.1 C. Figure 3g, and Figure S10 respectively show the GITT voltage curves of 1M, 0.2, 0.2Se, 0.2Te, and Se_0.15_Te_0.05_ during a single charge and discharge process. The nucleation and activation of Li_2_S can be represented by the immersion depth in the discharge and charge curves. The smaller the immersion depth, the higher the diffusion efficiency of Li^+^ [49]. In addition, the internal resistance can be obtained from the voltage changes of the electrodes during the intermittent titration process. As shown in the illustration of Figure 3g, V_0_ is the equilibrium voltage of the last relaxation. V_1_ represents the potential at the beginning of the pulse; V_2_ represents the potential at the end of the pulse, and V_3_ is the equilibrium voltage of the current relaxation. Based on the above data, use formula (2)

(2) $$D^{GITT}=\frac{4l^{2}}{\pi \tau }\;{\left(\frac{\Delta E_{s}}{\Delta E_{t}}\right)}^{2}\;$$

Here, D represents the lithium‐ion diffusion coefficient, l is the thickness of the electrode sheet, τ is the relaxation time, ΔE_s_ indicates the potential difference between the two equilibrium voltages (ΔEs = V_3_‐V_0_), and ΔE_t_ represents the potential difference between the start and end of charging/discharging (ΔE_t_ = V_2_ – V_1_). Combining formula (2), the lithium‐ion diffusion coefficient curve, as shown in Figure 3h, can be obtained. It can be seen that the overall LogD of Se_0.15_Te_0.05_ is relatively higher, and the fluctuation is relatively gentle. This is attributed to the synergistic effect of the dual active sites of Se and Te, which enables the controllable diffusion of LiPS. It not only ensures the utilization of active substances but also enhances the nucleation, activation, and transformation efficiency of Li_2_S. However, the diffusion coefficient of Se_0.15_Te_0.05_ slightly decreases in the 60–70% stage. This might be due to the fact that Se and Te simultaneously promote some side reactions in the transformation of LiPS during the catalytic process, resulting in a large amount of LiPS being adsorbed in a short period of time. But thanks to its own excellent kinetics, at 80% of the stage, these LiPS can be quickly reduced (deposited) and then still maintain a relatively high diffusion coefficient.

**FIGURE 4:**
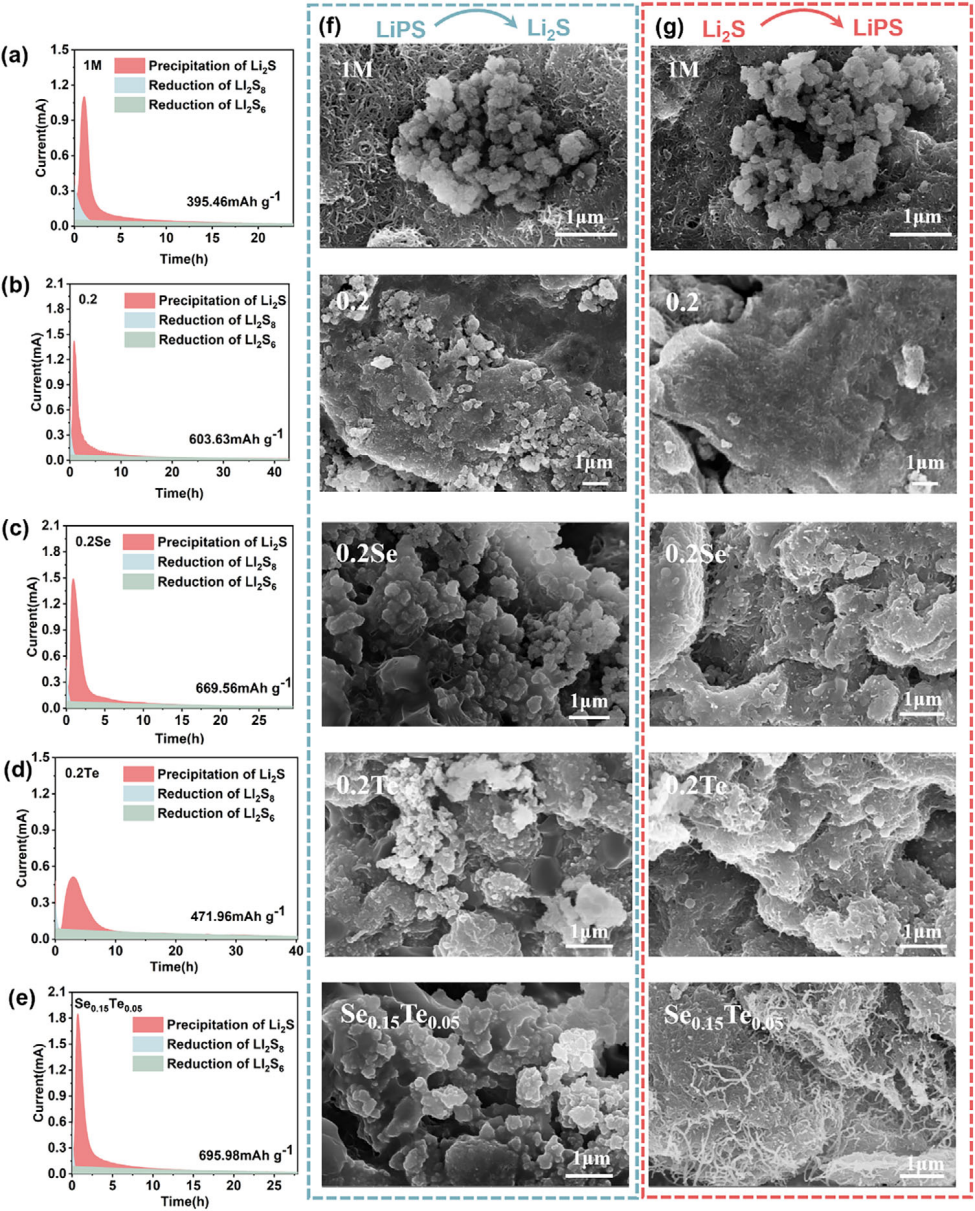
Li_2_S nucleation curves of (a) 1m, (b) 0.2, (c) 0.2Se, (d) 0.2Te, and (e) Se_0.15_Te_0.05_. Corresponding electrode morphologies (f) after Li_2_S nucleation and (g) dissolution to LiPS on the surface.

The nucleation and dissolution of Li_2_S were investigated through potentiostatic discharge experiments, thereby studying the catalytic effect of DPDSe‐Te generated by exchange at low concentrations on these processes [50]. Previous studies have indicated that when long‐chain LiPS are reduced to solid Li_2_S_2_/Li_2_S, these solid products tend to deposit on materials with high conductivity and strong affinity for LiPS, gradually forming a thick passivation layer [51, 52]. A comparison of the Li_2_S nucleation profiles in Figure 4a‐e reveals that the Se_0.15_Te_0.05_ cell delivered a Li_2_S deposition capacity of approximately 695.98 mAh g_s_^−1^, which is higher than those of the 1M (395.46 mAh g_s_^−1^), 0.2 (603.63 mAh g_s_^−1^), 0.2Se (669.56 mAh g_s_^−1^), and 0.2Te (471.96 mAh g_s_^−1^) cells. Furthermore, it also exhibited the highest current response, indicating rapid Li_2_S deposition kinetics. Owing to the similar formulation of 0.2Se and Se_0.15_Te_0.05_, their capacities were the closest. However, by incorporating DPDSe and DPDTe in the optimal proportion, the synergistic catalysis between the dual active sites of DPDSe‐Te achieved a higher deposition capacity and enhanced current response. Surface morphology analysis of the electrodes further elucidated the underlying reasons for the enhanced capacity. As shown in Figure 4f, inhomogeneous and bulk‐like Li_2_S deposition—driven by the inherent LiPS mediators—is observed in the 1M electrolyte. Upon reducing the electrolyte concentration, the 0.2 formulation, capable of dissolving more LiPS, results in a modest amount of Li_2_S deposited on the surface. The Se_0.15_Te_0.05_ electrolyte enables precise regulation of Li_2_S deposition, exhibiting the thickest yet uniformly smooth Li_2_S coating with complete surface coverage and a pronounced tendency toward three‐dimensional growth. This morphology suggests a deposition mechanism involving discrete nucleation and isotropic growth, wherein Li_2_S particles remain dispersed and isolated, thereby preserving surface activity. These findings confirm that the Se_0.15_Te_0.05_ electrolyte effectively improves reaction kinetics, enhances interaction with LiPS, increases sulfur utilization, and facilitates high‐capacity sulfur discharge.

Sustained galvanostatic discharge followed by potentiostatic charging allowed the deposited Li_2_S to be oxidized back into LiPS and re‐dissolved into the electrolyte. Comparison of the Li_2_S dissolution profiles in Figure S11 shows that the Se_0.15_Te_0.05_ electrolyte still exhibits the most pronounced dissolution capability and the highest oxidation current, suggesting superior oxidation capacity. As shown in Figure 4g, this observation is further supported by the morphological changes of the electrode surface after Li_2_S dissolution. In contrast to the other four cells, the solid deposition layer in the Se_0.15_Te_0.05_ cell is significantly dissolved, and the fibrous morphology of the CNT becomes visible again. This clearly indicates that Se_0.15_Te_0.05_ effectively catalyzes the oxidation of Li_2_S.

**FIGURE 5:**
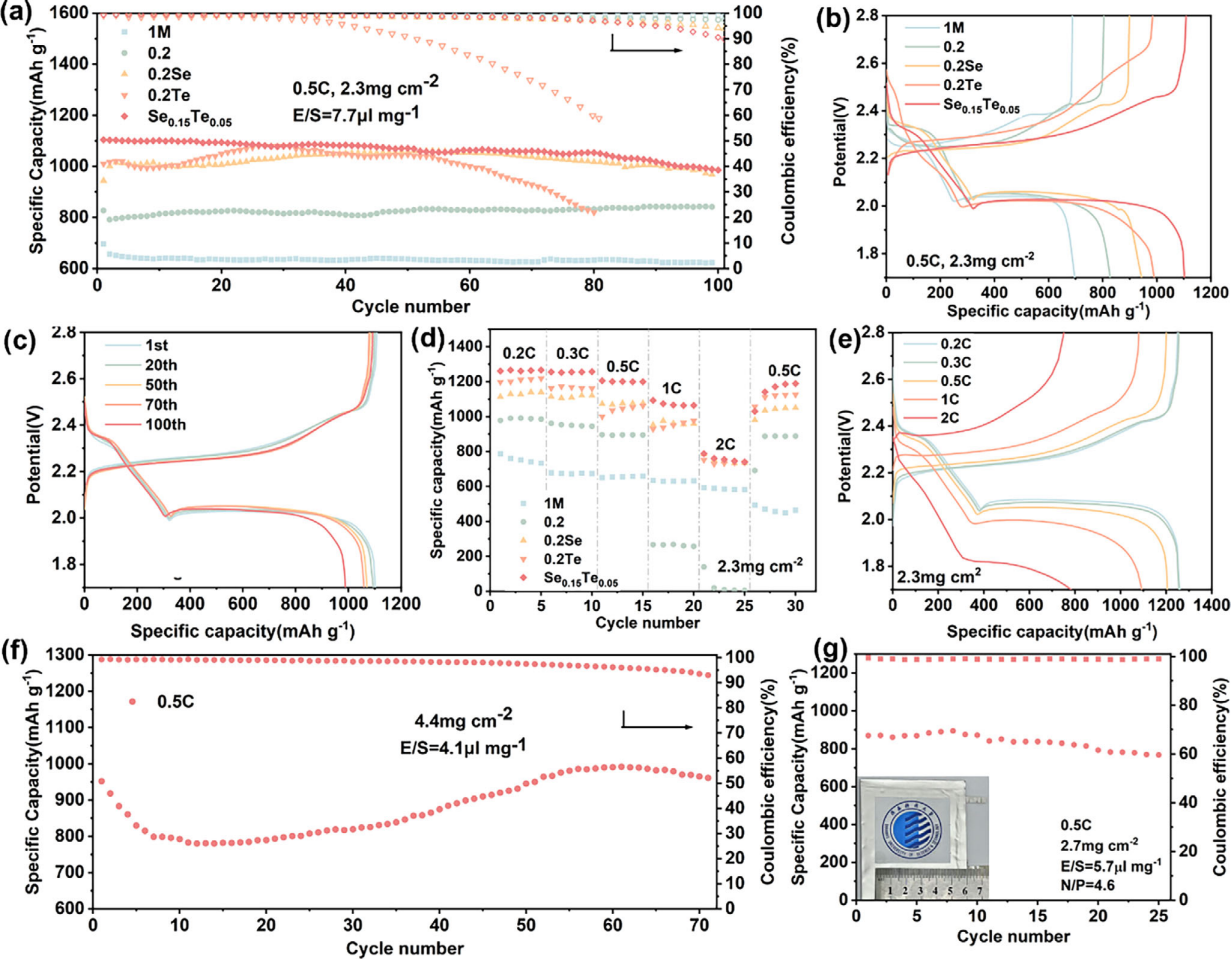
(a) Cycling performance of Li–S batteries using 1M, 0.2, 0.2Se, 0.2Te, and Se_0.15_Te_0.05_ electrolytes at 0.5 C. (b) The third‐cycle charge–discharge curves of Li–S batteries with five electrolytes at 0.5 C. (c) Charge–discharge curves of Se_0.15_Te_0.05_ battery at different cycles. (d) Rate performance and (e) corresponding charge/discharge curves of the five Li–S batteries. (f) Cycling performance of Se_0.15_Te_0.05_ Li–S battery under a high sulfur loading of 4.4 mg cm^−2^ and an E/S ratio of 4.1 µL mg^−1^. (g) Cycling performance of Li–S pouch cell mediated by Se_0.15_Te_0.05_ with a high sulfur loading of 2.7 mg cm^−2^ and an E/S ratio of 5.7 µL mg^−1^ at 0.5 C.

To further investigate the electrochemical performance of the additives in Li–S batteries, cathodes were prepared via a coating method (sulfur content > 60 wt.%, areal loading of 2.3 mg cm^−2^) and paired with lithium metal anodes for cell assembly. As shown in Figure 5a, at a rate of 0.5 C, the Se_0.15_Te_0.05_ cell delivered a high initial specific capacity of 1103 mAh g^−1^, corresponding to a sulfur utilization of 65.9%. After 100 cycles, it still maintained a specific capacity of 985 mAh g^−1^, with a capacity retention rate of 89.3%. The Se_0.15_Te_0.05_ cell shows a late‐stage coulombic efficiency decline. A low‐salt electrolyte and Se/Te additives are employed to balance high sulfur utilization with cycling stability; therefore, achieving ∼100 stable cycles under high sulfur loading is considered meaningful. As can be seen from Figure 5b, compared with the other four electrolytes, the Se_0.15_Te_0.05_ battery exhibits a higher specific capacity. In addition, the charge–discharge voltage curves of the first cycle can clearly identify two characteristic discharge platforms of 2.4 V (Li_2_S_6_ to Li_2_S_4_) and 2.0 V (Li_2_S_4_ to Li_2_S_2_/Li_2_S), as well as one charging platform, which are in good agreement with the two‐step lithiation of sulfur and its reversible oxidation reaction, respectively. In Figure 5b, 0.2Se exhibits a slightly lower apparent polarization compared with Se_0.15_Te_0.05_. Because Se_0.15_Te_0.05_ delivers a higher reversible capacity, the reaction proceeds deeper into the late‐stage conversion where Li_2_S_2_ and Li_2_S deposition and mass‐transport limitations become more pronounced, leading to a larger apparent polarization. Notably, EIS and ionic‐conductivity results indicate lower resistance and faster kinetics for Se_0.15_Te_0.05_, which is also consistent with its smaller polarization gap at higher rates (1 C) (Figure S12).

Moreover, as corroborated by Figure 5c, even at the 100th cycle, the voltage plateaus of the Se_0.15_Te_0.05_ cell remain significantly more stable in each stage. This enhanced stability is primarily attributed to the ability of Se_0.15_Te_0.05_ to facilitate the participation of a greater fraction of LiPS in the reactions, which improves sulfur utilization and thereby increases the overall cell capacity. To evaluate the performance of the Li–S batteries under high C‐rates, tests were conducted as shown in Figure S12. At 1 C, the Se_0.15_Te_0.05_ cell also delivered an initial specific capacity of 943 mAh g^−1^. Under the same conditions, the other four cells exhibited significant polarization when the C‐rate was increased, whereas the Se_0.15_Te_0.05_ cell demonstrated a smaller polarization voltage gap. This indicates that the synergistic effect of DPDSe‐Te at low concentrations enhances the electrocatalytic activity, resulting in earlier anode and cathode reactions during charging and discharging [53]. As shown in Table S1, compared with representative previously reported Se/Te‐related electrolyte additive studies, Se_0.15_Te_0.05_ exhibits excellent electrochemical performance. These results indicate that a low‐concentration synergistic Se/Te co‐additive strategy can effectively accelerate the reaction kinetics of LiPS, delivering performance superior to or comparable with most analogous electrolyte systems.

In addition, the long‐cycle performance of the Se_0.15_Te_0.05_ battery was further evaluated. As shown in Figure S13, after 150 cycles at 0.5 C, its specific capacity remains at 942.2 mAh g^−1^, with a capacity retention rate of 87.1%. To quantitatively evaluate the potential contribution of the additives themselves to the battery capacity, symmetric cells were assembled using sulfur‐free electrodes and tested with the five electrolytes. As shown in Figure S14, the 0.2Se electrolyte contributed a specific capacity of 92.6 mAh g^−1^, the 0.2Te electrolyte contributed 60.2 mAh g^−1^, while the Se_0.15_Te_0.05_ hybrid electrolyte contributed only 70.4 mAh g^−1^. This value is significantly lower than the actual cell capacity, further confirming that the excellent electrochemical performance of the Se_0.15_Te_0.05_ cell shown in Figure 5 primarily stems from the highly efficient synergistic catalytic effect of the additives on polysulfide conversion rather than its own capacity contribution.

Figure 5d further illustrates the rate capabilities of these cells. The Se_0.15_Te_0.05_ cell outperformed the other five cells across all tested rates. Although the specific capacity decreased with increasing C‐rate, it still reached 783 mAh g^−1^ at 2 C. Notably, when the C‐rate was restored to 0.5 C, the discharge capacity recovered effectively, demonstrating excellent reversibility and rate performance. The charge–discharge curves at different C‐rates shown in Figure 5e also highlight the advantages of mixing DPDSe and DPDTe additives at low concentrations in enhancing reaction kinetics.

The superiority of Se_0.15_Te_0.05_ was further demonstrated under conditions of high areal sulfur loading and lean electrolyte. Figure 5f shows the cycling performance of cells with a high sulfur loading of 4.4 mg cm^−2^ and a lean electrolyte volume (E/S = 4.1 µL mg^−1^). The Se_0.15_Te_0.05_ cell retained a capacity of 965 mAh g^−1^ after 70 cycles at a high C‐rate of 0.5 C, reaffirming its excellent rate capability. The initial decrease in discharge specific capacity, followed by a gradual increase, may be attributed to incomplete activation of sulfur on the cathode during early cycles under high C‐rate, where the full catalytic activity had not yet been realized. To validate the applicability of Se_0.15_Te_0.05_ in scenarios demanding high energy density, pouch cells were assembled and evaluated with a sulfur loading of 2.7 mg cm^−2^, a Negative/Positive (N/P) ratio of 4.6, and an E/S ratio of 5.7 µL mg^−1^. As shown in Figure 5g and Table S2, the pouch cell delivered an initial specific capacity of 869 mAh g^−1^ at 0.5 C, corresponding to an energy density of 340 Wh kg^−1^. After 25 cycles, it maintained a specific capacity of 767 mAh g^−1^ with a capacity retention of 88.3%, equivalent to a high retained energy density of 300 Wh kg^−1^. Future work will focus on enabling lean‐electrolyte operation through electrolyte formulation optimization, reducing anode excess while maintaining interfacial stability, and scaling toward multilayer pouch cell configurations.

Through the comparison of battery performance with different concentration ratios in Figure S15, the superiority of the Se_0.15_Te_0.05_ cell is clearly demonstrated. The control group Se_0.01_Te_0.01_ cell delivered only an initial specific capacity of 745 mAh g^−1^ [32], significantly lower than the 1103 mAh g^−1^ achieved by the Se_0.15_Te_0.05_ cell. Moreover, the Se_0.15_Te_0.05_ cell exhibited notable advantages in both rate capability and long‐term cycling stability. These comparison results strongly indicate that low‐concentration conditions can enable more sulfur to participate in the reaction. Additionally, the Se_0.15_Te_0.05_ electrolyte formula maximizes the synergistic catalytic effect of the dual active sites in DPDSe and DPDTe, which is a key proportion for achieving the best battery kinetic performance.

Analysis of the electrode structure and lithium anode stability further elucidates its advantages. Previous studies have indicated that the continuous solid–liquid conversion between S_8_, LiPS, and Li_2_S tends to cause redistribution of sulfur on the electrode surface and deposition of inert sulfides, forming an insulating passivation layer. This degrades the electroactive surface, leading to sluggish kinetics and electrode deactivation [54]. To evaluate the catalytic stability, Figure S16 selected and compared the SEM morphology of the cathode of 1M and Se_0.15_Te_0.05_ cells before and after 100 cycles at a sulfur loading of 2.3 mg cm^−2^ and a rate of 0.5 C. While the pre‐cycling morphology was similar for both, after cycling, the 1M cell electrode surface was heavily covered and clogged with large deposits, indicating severe degradation of catalytic surface activity and impaired effective contact between LiPS and the electrode. In contrast, the fibrous structure of CNT remained clearly visible on the cycled Se_0.15_Te_0.05_ electrode, demonstrating significantly reduced passivation layer formation. This provides strong evidence that the Se_0.15_Te_0.05_ additive offers exceptional catalytic stability, effectively maintaining electrode structural integrity and active surface availability over extended cycling. On the other hand, the aggregation and reduction of LiPS on the surface of lithium metal anodes are the key factors leading to the corrosion deterioration of lithium anodes [39]. As shown in Figure S17, under the same cycling conditions (0.5 C, 100 cycles), the surface of the lithium metal anode using Se_0.15_Te_0.05_ electrolyte is relatively smooth, and the damage is significantly reduced. In contrast, the lithium anodes of other batteries exhibit obvious cracks and a rough surface with grooves, indicating that the Se_0.15_Te_0.05_ additive can efficiently catalyze LiPS, significantly reducing the corrosion of LiPS on the lithium anode. It shows good chemical stability over long‐term cycling. These results once again strongly confirm the outstanding performance of DPDSe‐Te, which is generated by regulating the ratio of DPDSe/DPDTe additives at low concentrations, with its dual active sites and synergistic catalysis in enhancing the redox kinetics of Lithium–sulfur batteries and maintaining the stability of the electrode interface, making it an ideal electrolyte for achieving high‐performance Lithium–sulfur batteries.

## 4 Conclusion

In summary, this study proposes a dual‐strategy electrolyte engineering method at low concentrations to address the key challenges of low solubility and slow kinetics in Li‐S batteries. By synergistically coupling low‐concentration electrolytes with an optimized DPDSe/DPDTe additive formula, significant improvements were achieved in the utilization rate of active substances and high‐rate performance. Compared with the commercial electrolyte (1M) and other low‐concentration electrolytes (0.2, 0.2Se, 0.2Te), the optimized mixed additive system (Se_0.15_Te_0.05_) further reconstructs the solvation structure via DPDSe‐Te interactions, exhibits the highest sulfur solubility, and enhances the accessibility of active materials. At the same time, by taking advantage of its synergistic catalytic effect, the transformation kinetics of LiPS were significantly accelerated. Electrochemical tests confirmed that the DPDSe and DPDTe additives, as dual active sites, promoted the continuous sulfur redox reaction, achieving efficient deposition and dissolution of Li_2_S. The cell employing the Se_0.15_Te_0.05_ electrolyte delivered an initial specific capacity of 1103 mAh g^−1^ (65.9% sulfur utilization) at 0.5 C and retained 89.3% of its capacity after 100 cycles. Even at a high rate of 2 C, a capacity of 783 mAh g^−1^ was maintained. In addition, the Se_0.15_Te_0.05_ electrolyte enables the Li‐S pouch cell to deliver an energy density of 340 Wh kg^−1^. This work establishes a new paradigm for electrolyte design in multi‐electron transfer battery systems, offering a feasible pathway toward high‐power and high‐energy‐density Li–S batteries without compromising the inherent advantages of low‐concentration electrolytes, such as low viscosity and low cost. Future work will focus on further optimizing the electrolyte formulation to enable stable operation under leaner electrolyte conditions (lower E/S ratios) and on evaluating long‐term cycling stability, which will help bridge the gap between fundamental research and practical applications.

## Author Contributions

**Lin Zhang** and **Haiwei Wu** conceived and supervised the project and jointly provided financial support. **Ruihua Li** and **Hairu Wei** designed and performed the experiments, analyzed and interpreted the data, and wrote the original manuscript. **Zhihua Lin**, **Frederik Bettels**, **Leon Schenk** and **Wenhao Jia** assisted in laboratory management. **Zhijian Li**, **Hanbin Liu** and **Guodong Liu** also supervised the project and helped to revise the manuscript.

## Conflicts of Interest

The authors declare no conflicts of interest.

## Supporting information




**Supporting File**: advs75377‐sup‐0001‐SuppMat.docx.

## Data Availability

The data that support the findings of this study are available on request from the corresponding author. The data are not publicly available due to privacy or ethical restrictions.
