# Supplementary material for: Enhancing Li‐S Battery Performance Through Low‐Concentration Electrolytes with Organic Se/Te Co‐Additives to Address Solubility and Kinetic Challenges
Source: Adv Sci (Weinh). 2026 Apr 24:e75377. Online ahead of print. doi: 10.1002/advs.75377 (PMC13334614; doi:10.1002/advs.75377)
Supplement: Supplementary file 1 — Supporting File: advs75377‐sup‐0001‐SuppMat.docx. [file ADVS-9999-e75377-s001.docx]

**Supporting Information**

**Enhancing Li-S Battery Performance through Low-Concentration Electrolytes with Organic Se/Te Co-Additives to Address Solubility and Kinetic Challenges**

Ruihua Li^a, 1^, Haiwei Wu^a, b,^ ^[[1]](#footnote-1)^, Hairu Wei^b, 1^, Wenhao Jia^a^, Frederik Bettels^b^, Leon Schenk^b^, Zhihua Lin^b^, Hanbin Liu^a^, Guodong Liu^a^, Zhijian Li^a, *^, Lin Zhang ^b, *^

^a^ College of Bioresources Chemical and Materials Engineering, Shaanxi University of Science & Technology, Xi’an 710021. China

^b^ Institute for Solid State Physics, Leibniz Universitat Hannover Fakultat fur Mathematik und Physik, Appelstrasse 2, Hannover 30167, Germany

^1^ Ruihua Li and Hairu Wei contributed equally to this work

**Experimental Section**

**Assembly of lithium-sulfur batteries.** Under an argon atmosphere, the prepared positive electrode sheet was cut into a diameter of 12 mm and used as the positive electrode of the battery. The negative electrode is a lithium sheet, and Celgard 2500 microporous PP film is used as the battery separator. Place the positive electrode sheet at the center of the positive electrode shell of the CR2016 button battery, add 20 μL of electrolyte, quickly cover the separator on the positive electrode material to ensure the separator is fully soaked, and place the lithium sheet at the center of the negative electrode shell. Drop 10 μL of electrolyte, attach the negative electrode shell to the positive electrode shell, and then put it into the hydraulic button battery sealing machine for sealing.

**Assembly of symmetrical batteries.** Symmetrical batteries were constructed using CR2016 button batteries in an argon atmosphere. The symmetrical battery was assembled by using two sulfur-free carbon paper electrodes (with a diameter of 12 mm) as the cathode and anode, Celgard 2500 membrane as the separator, and five 30 μL 0.5M Li_2_S_8_ electrolytes as the electrolyte. The CV curve of symmetrical batteries operates within the voltage range of -1.5 to 1.5V (relative to Li^+^/Li). The EIS was tested by the Chenhua Electrochemical Workstation (CHI760E), with a frequency range of 0.01Hz to 100kHz.

**Supporting tables**

**Tab.S1.** Comparison of Li-S battery performance using Se_0.15_Te_0.05_ versus reported Se/Te-based electrolyte additives.

| **Electrolyte** | **Sulfur loading** | **Current density** | **Initial specific capacity**  **(mAh g^-1^)** | **Cycle performance**  **(Capacity retention rate)** | **References** |
| --- | --- | --- | --- | --- | --- |
| Se_0.15_Te_0.05_ | 2.3mg cm^-2^ | 0.5C | 1103 | 89.3%(100cycles) | **This Work** |
| Mixed-Se | 1.2 mg cm^-2^ | 0.5C | 956 | 81.6%(200cycles) | [1] |
| Mixed-Se/Te | 1.5 mg cm^-2^ | 0.2C | 1350 | 81%(100cycles) | [2] |
| PTA-Se | 1.0-1.5 mg cm^-2^ | 0.2C | 1319 | 76%(150cycles) | [3] |
| With DPDTe | 1.2 mg cm^-2^ | 0.5C | 1227.3 | 52.06%(300cycles) | [4] |
| 0.7M PhSeH | 1.1 mg cm^-2^ | 0.5C | 1436 | 91.86%(200cycles) | [5] |
| PDSe | 1.0 mg cm^-2^ | 0.5C | ＜800 | 79.6%(200cycles) | [6] |

**Tab.S2.** Mass of each component used for pouch cell performance calculations (electrolyte, anode, sulfur cathode, and separator), and the corresponding calculated energy density.

| Component | Mass (mg) |
| --- | --- |
| Electrolyte | 310 |
| Anode | 107 |
| Cathode (S) | 88.4 |
| Separator | 44 |
| Total | 549.4 |
| Energy density | 340 Wh kg^-1^ |

**Supporting figures**


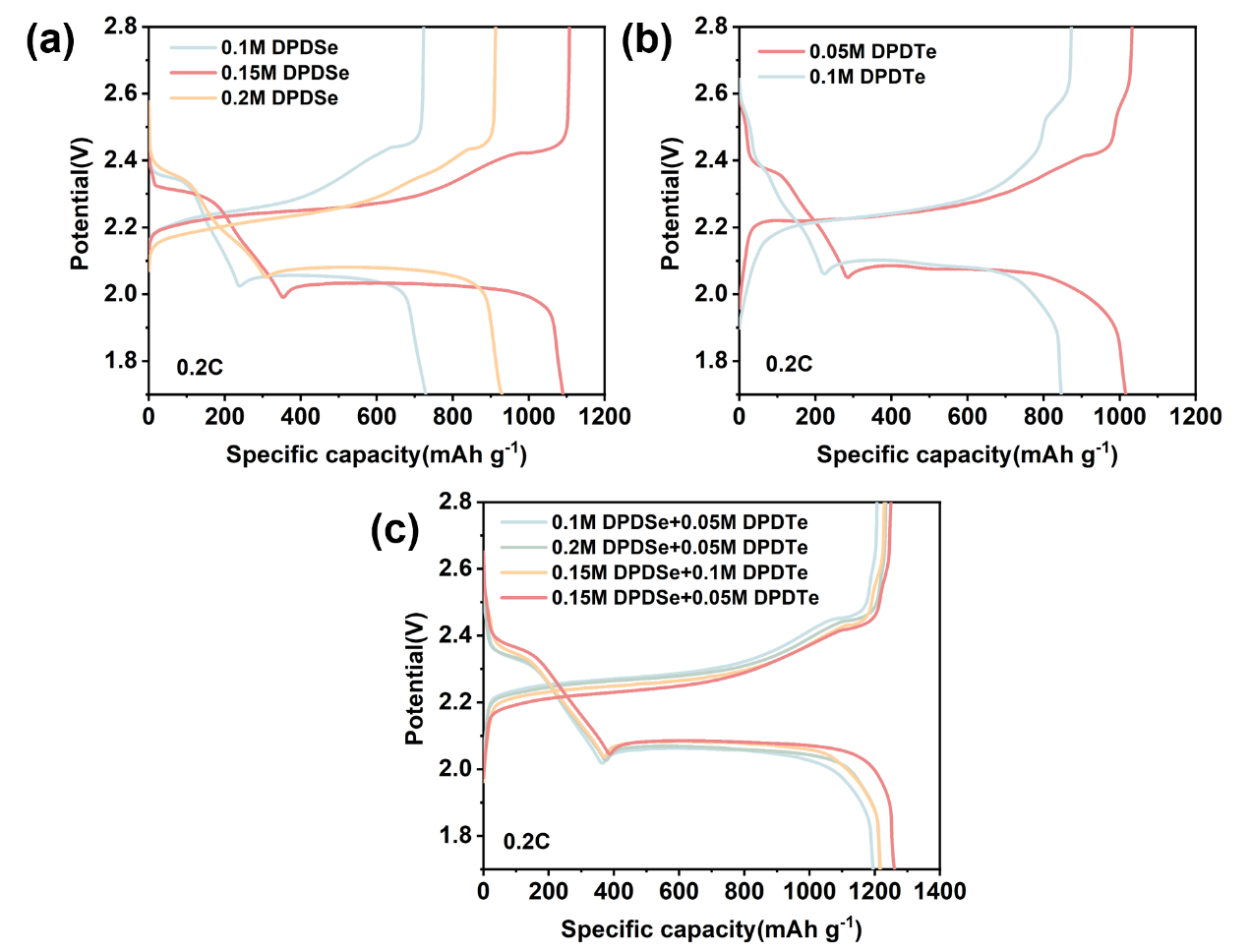


**Fig. S1.** The third cycle charge-discharge curves of electrolytes with different concentrations of (a) DPDSe, (b) DPDTe, and (c) their mixing ratio at 0.2 C.


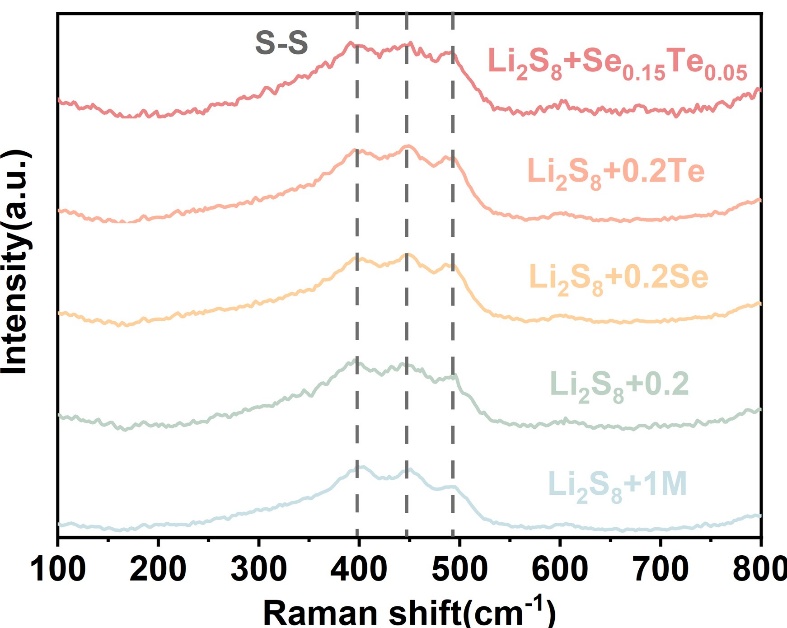


**Fig.S2.** Raman spectra of Li_2_S_8_ in 1M, 0.2, 0.2Se, 0.2Te, and Se_0.15_Te_0.05_.


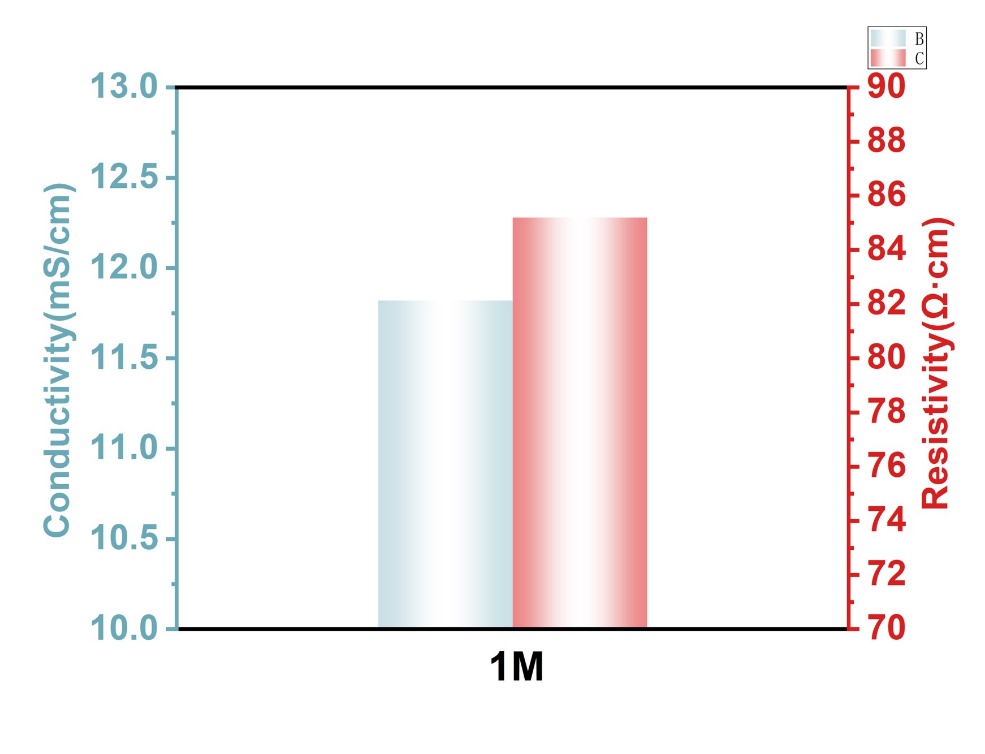


**Fig.S3.** Ionic conductivity of 1M.


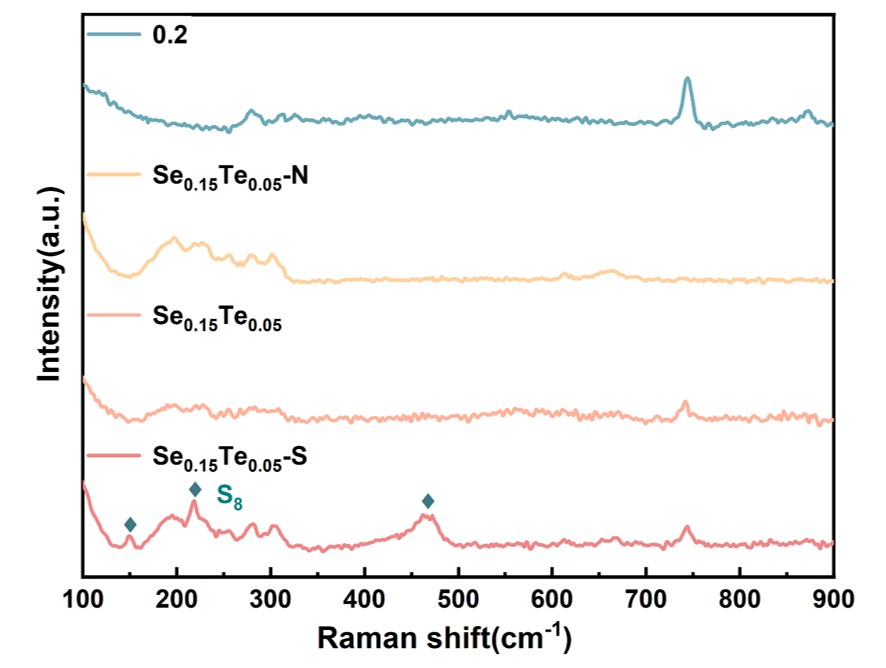


**Fig.S4.** Raman spectra of sulfur powder added to Se_0.15_Te_0.05_.


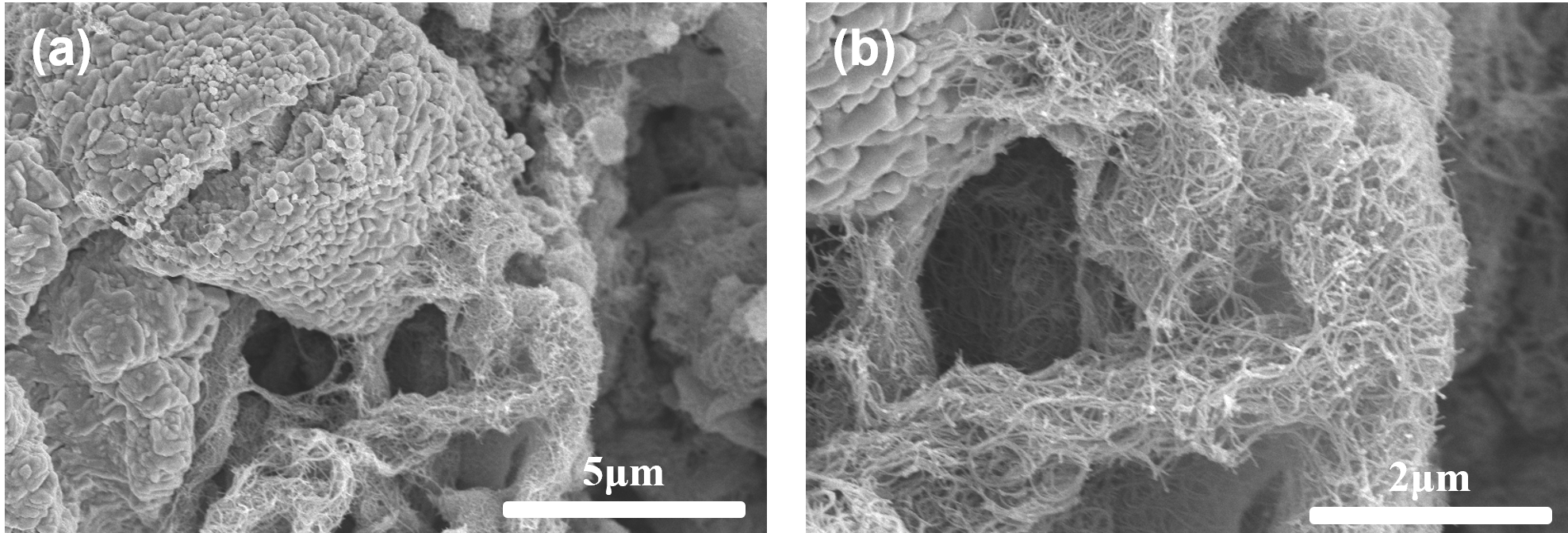


**Fig.S5.** SEM images of electrode powder.


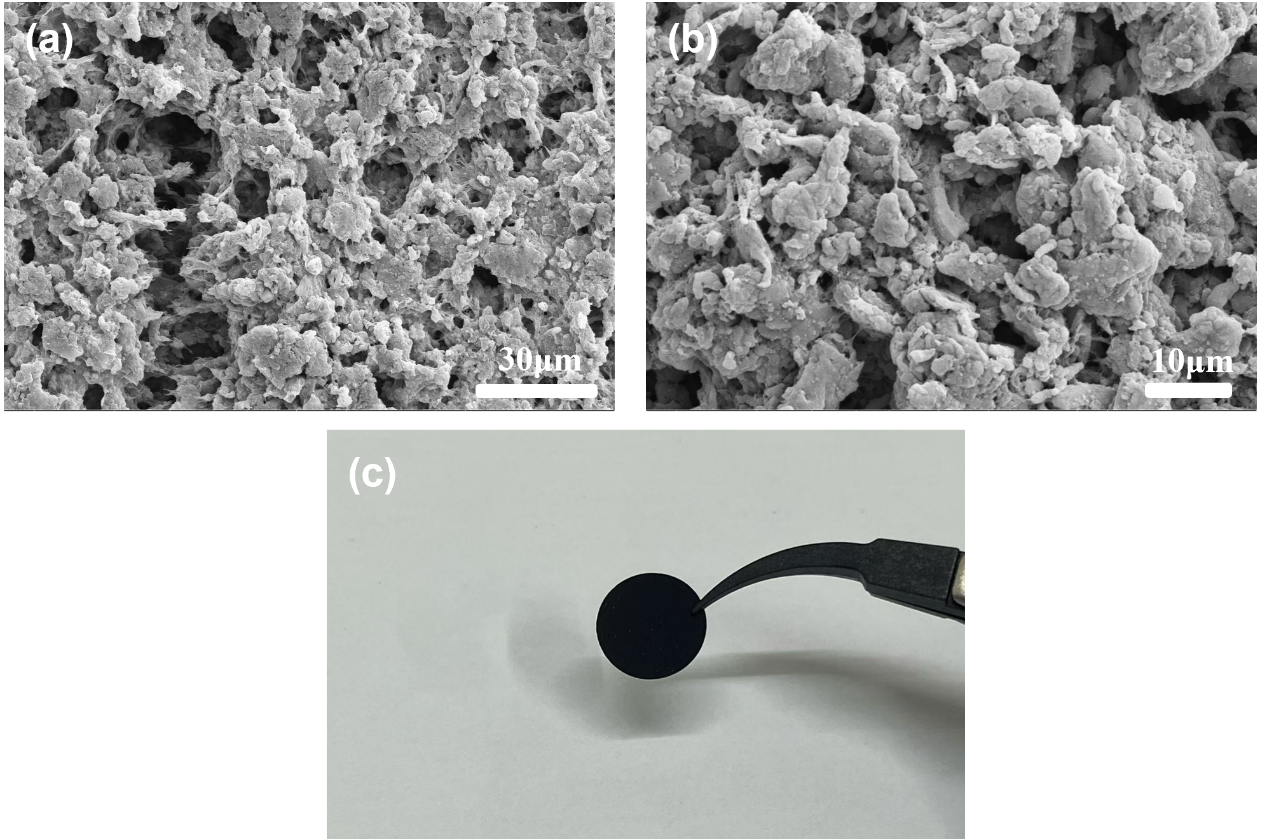


**Fig.S6.** (a) low-magnification SEM image, (b) high-magnification SEM image and (c) the photograph of the electrode sheet.


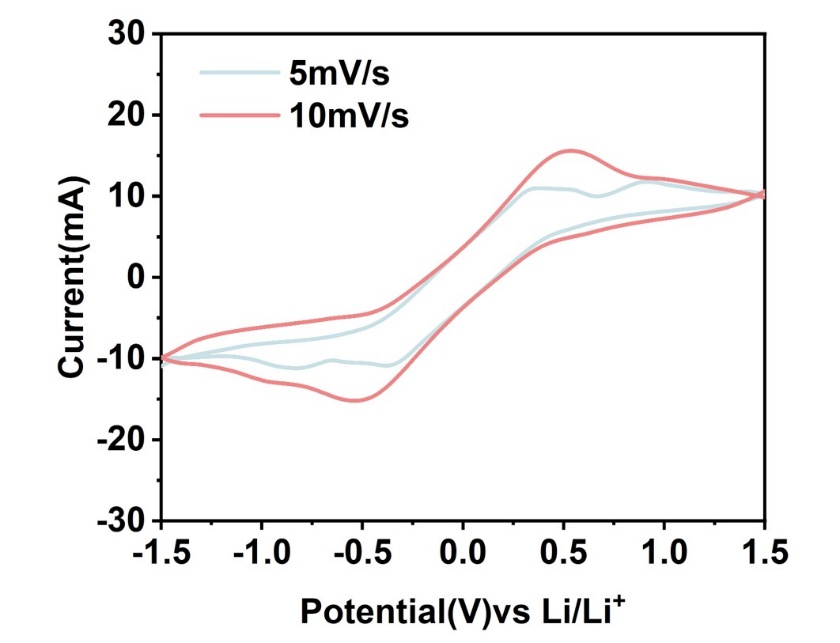


**Fig.S7.** CV curves at 5 mV s^-1^ and 10 mV s^-1^ for symmetric cells containing Li_2_S_8_ in Se_0.15_Te_0.05_ electrolytes.


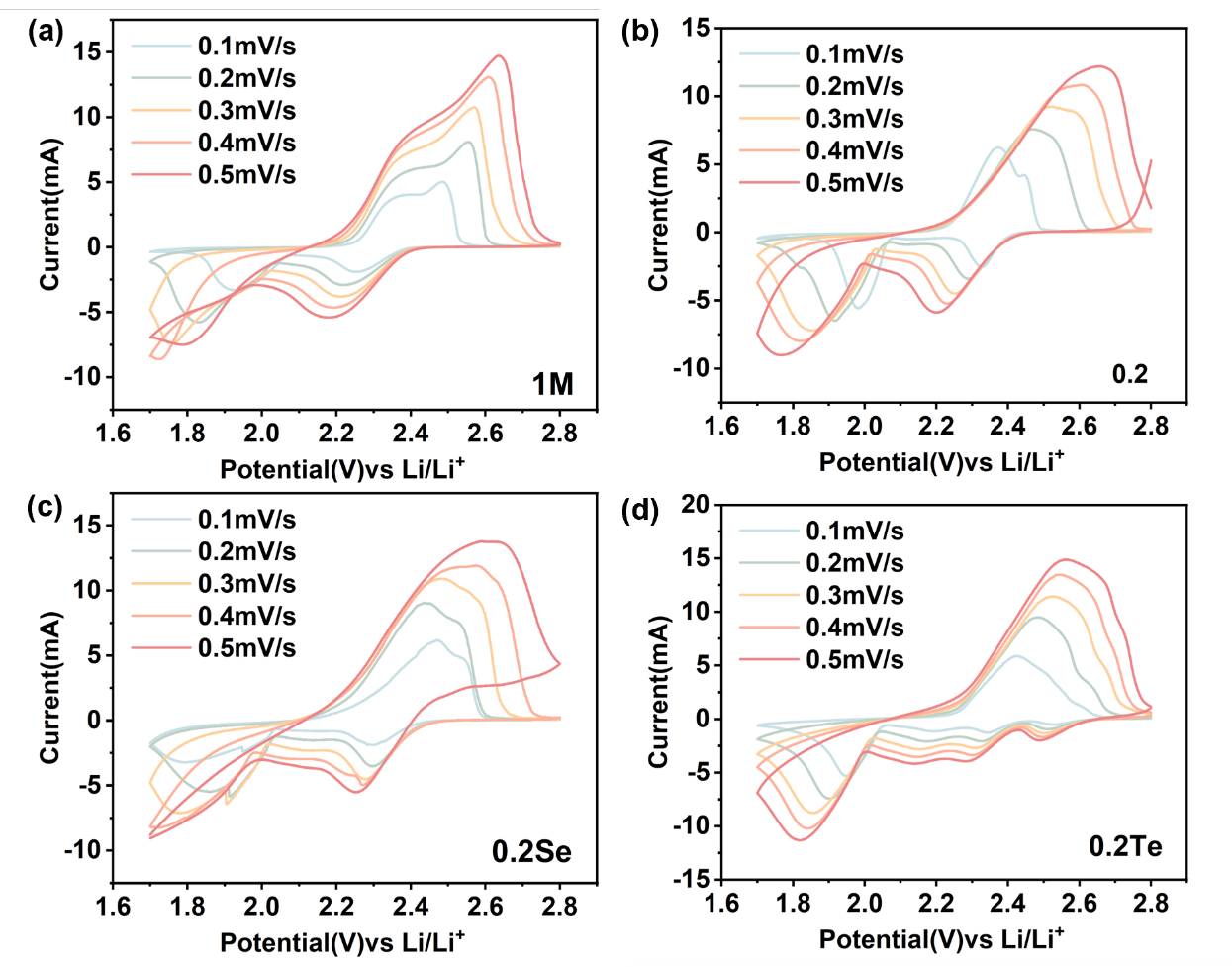


**Fig.S8.** CV curves of the (a) 1M, (b) 0.2, (c) 0.2Se and (d) 0.2Te cells at scan rates from 0.1 to 0.5 mV s^-1^.


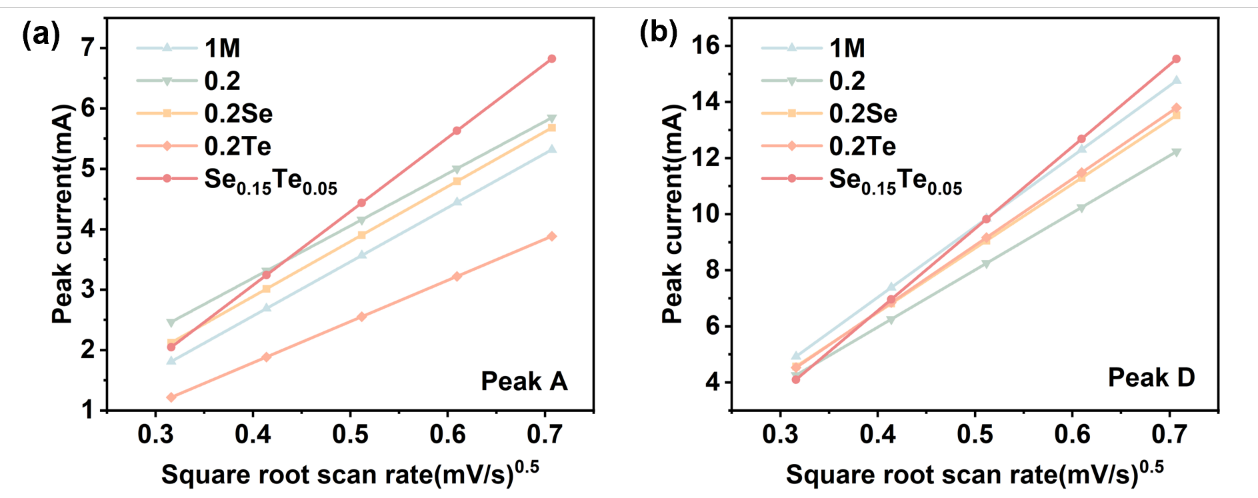


**Fig.S9.** CV peak currents of (a) peak A and (b) peak D versus the square root of the scan rate.


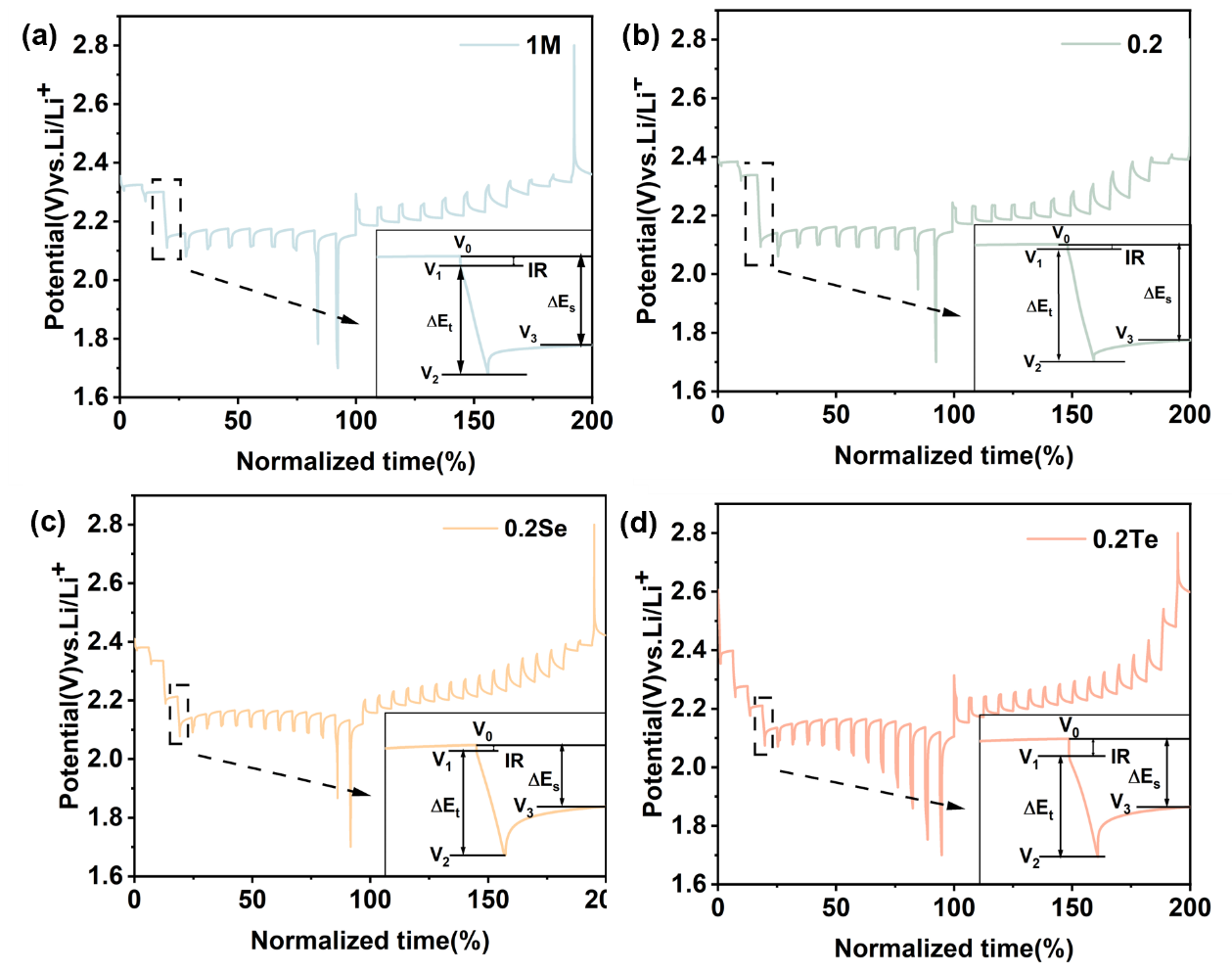


**Fig.S10.** GITT curves and their local magnification for the (a) 1M, (b) 0.2, (c) 0.2Se and (d) 0.2Te batteries.


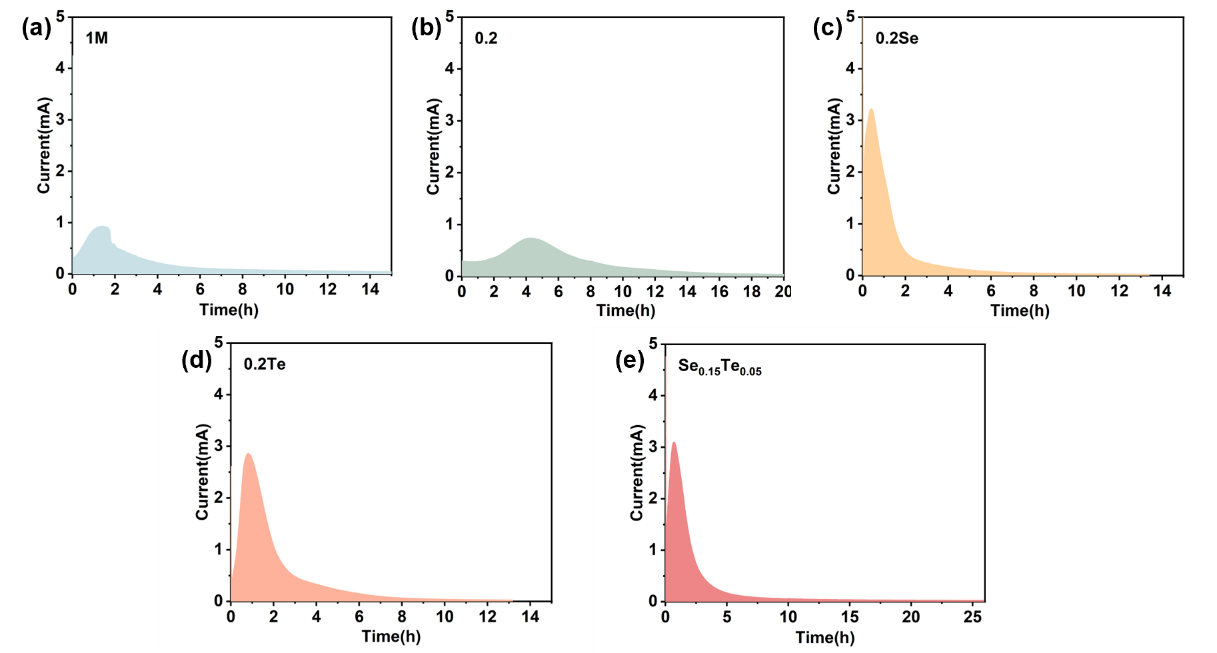


**Fig.S11.** Li_2_S dissolution curves of (a) 1M, (b) 0.2, (c) 0.2Se, (d) 0.2Te, and (e) Se_0.15_Te_0.05_.


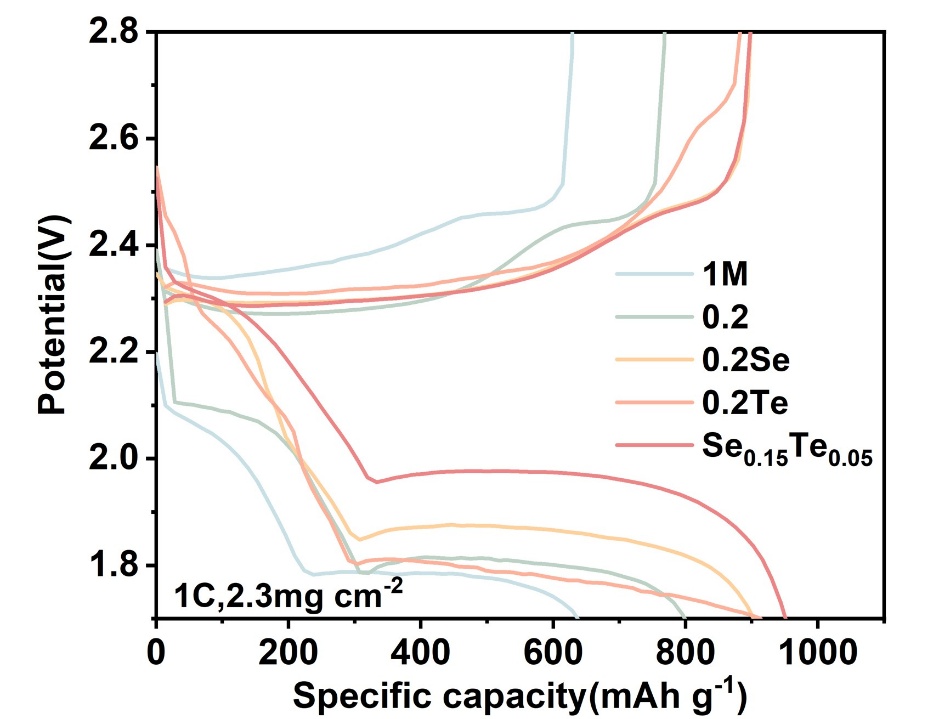


**Fig.S12.** The third-cycle charge-discharge curves of Li-S cells with five electrolytes at 1 C.


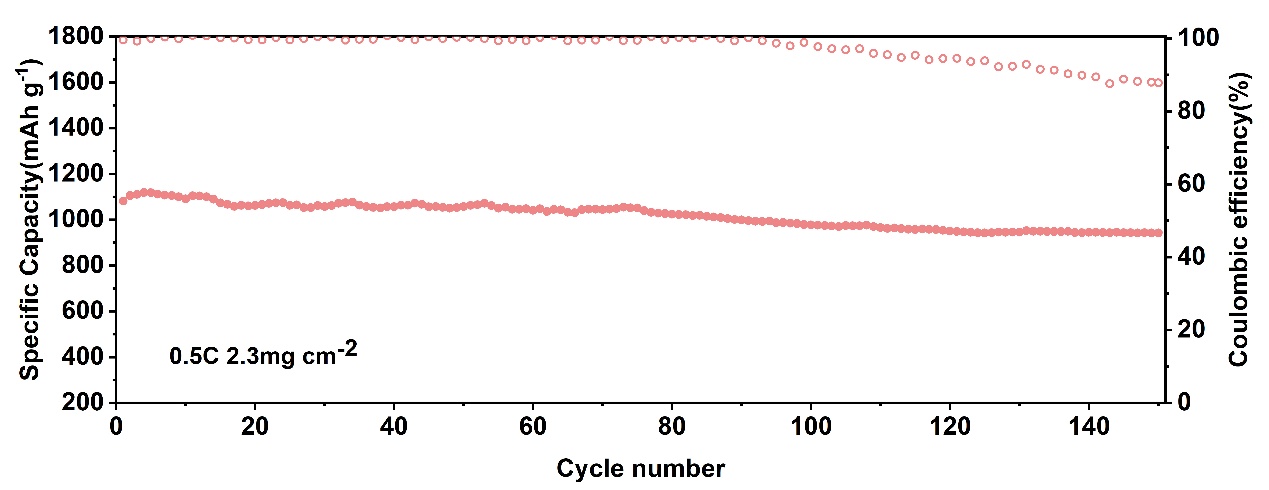


**Fig.S13.** Long-cycle performance of Li-S battery using Se_0.15_Te_0.05_ electrolyte at 0.5 C.


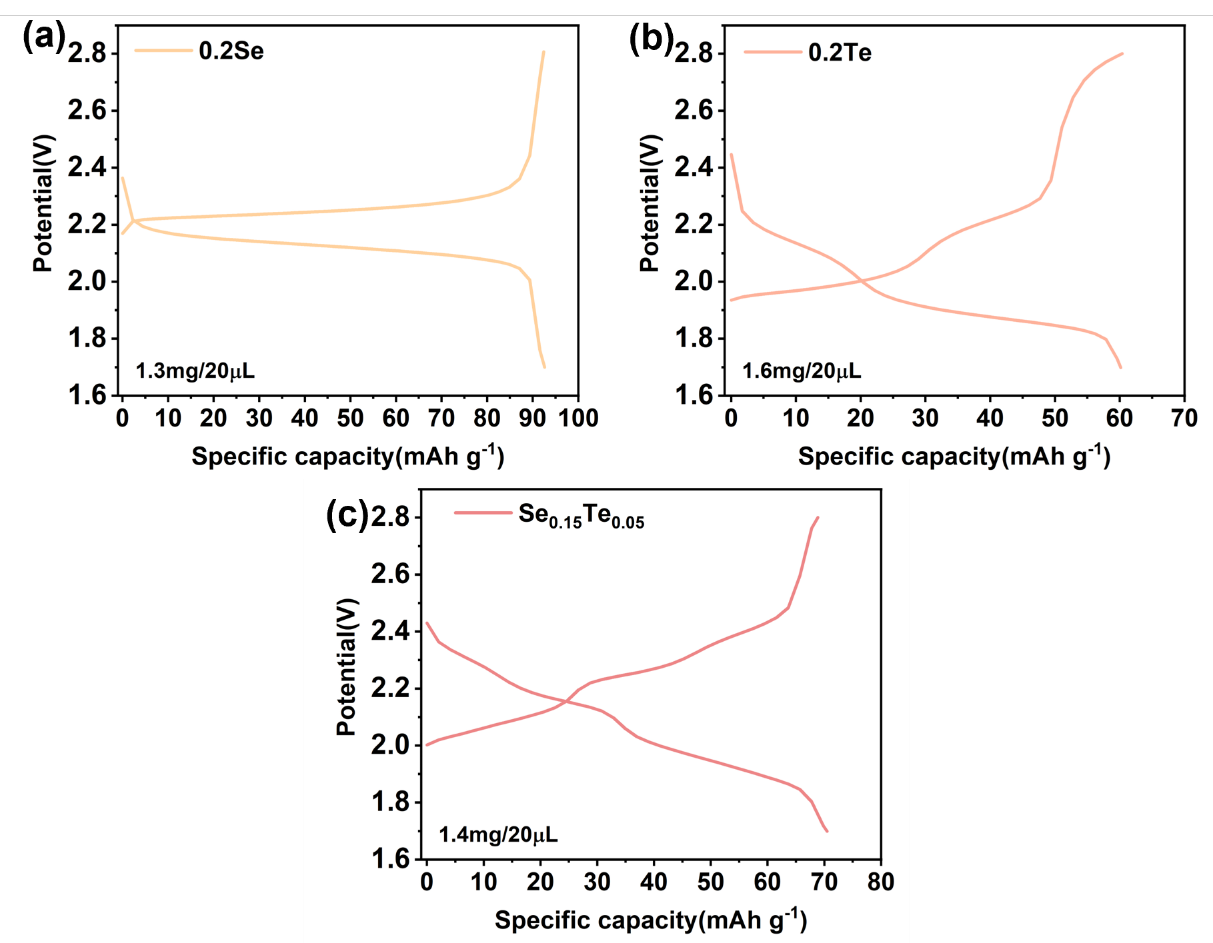


**Fig.S14.** The charge-discharge curves of symmetric cells assembled with sulfur-free electrodes and (a) 0.2Se, (b) 0.2Te, and (c) Se_0.15_Te_0.05_ electrolytes.


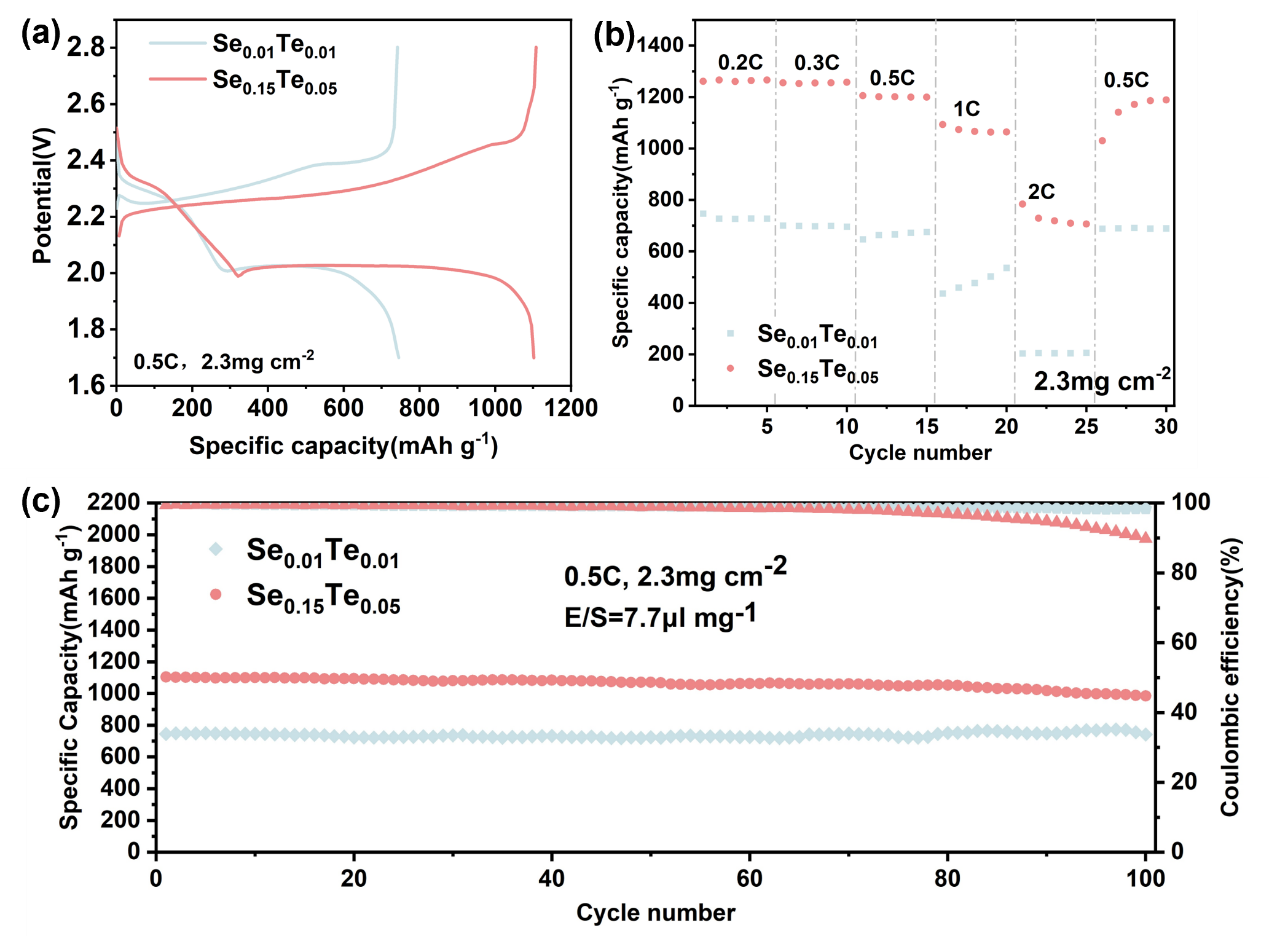


**Fig.S15.** (a) The third-cycle charge-discharge curves at 0.5 C, (b) rate capability, and (c) cycling performance at 0.5 C for Li-S cells assembled with Se_0.15_Te_0.05_ and Se_0.01_Te_0.01_ electrolytes.


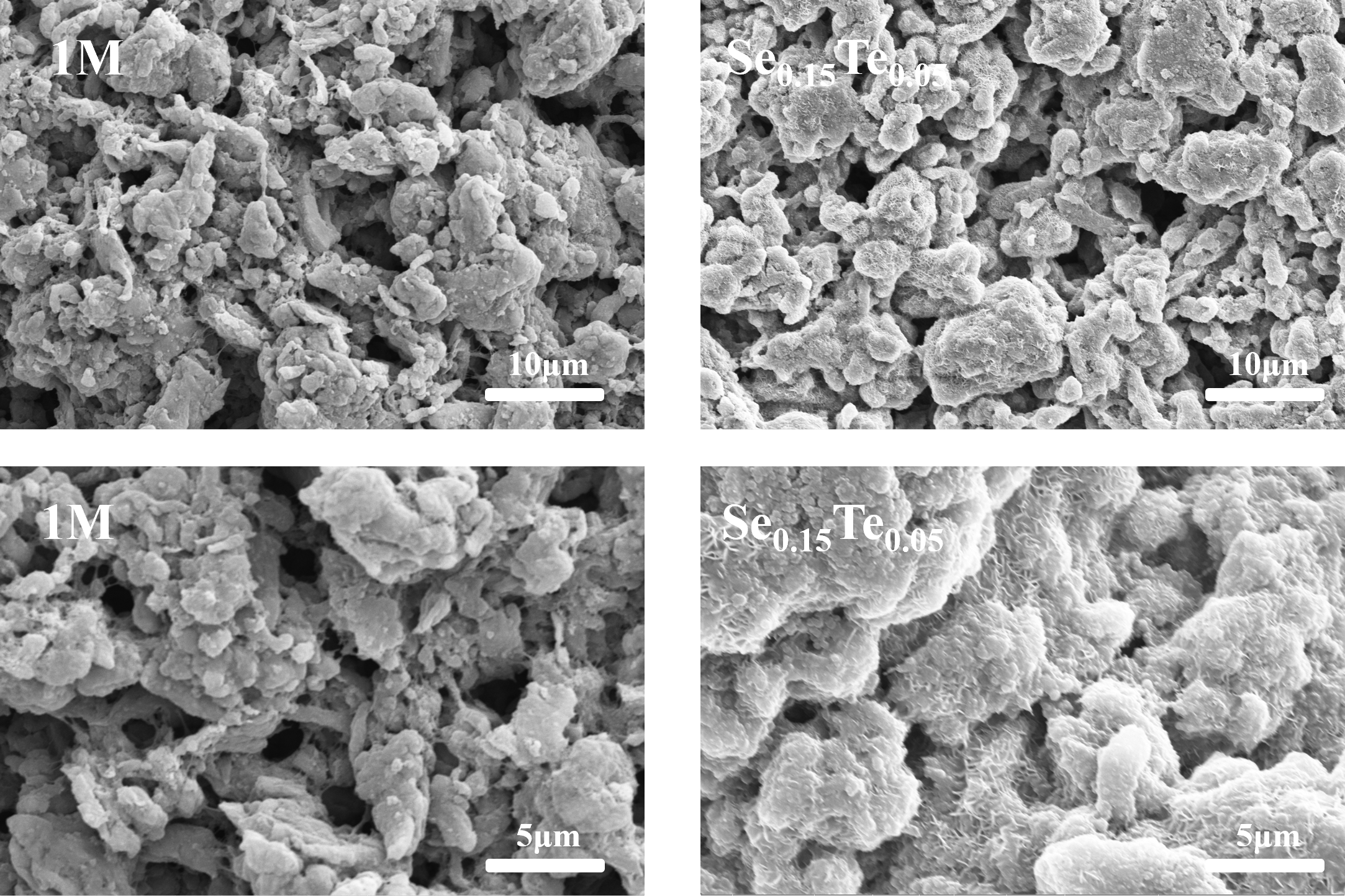


**Fig.S16.** SEM images of the electrodes from cells using the 1 M and the Se_0.15_Te_0.05_ electrolyte before and after 100 cycles.


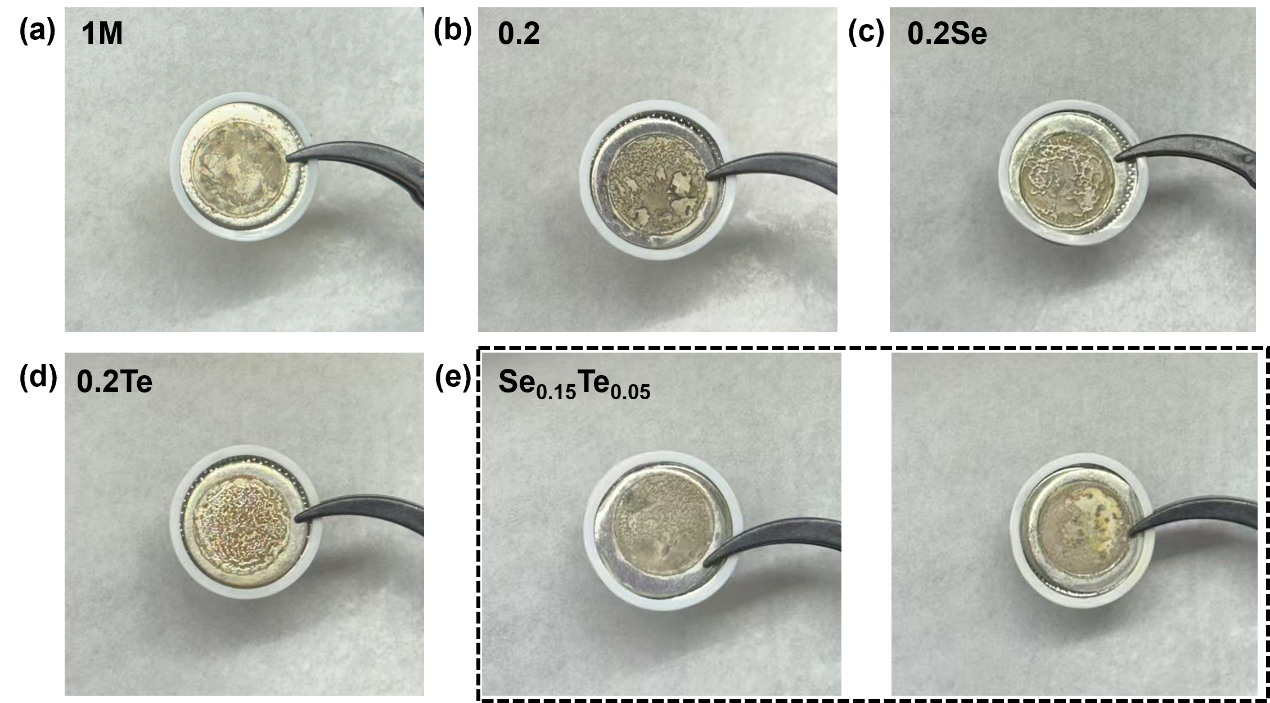


**Fig.S17.** Photographs of the lithium sheet surfaces from cells using (a) 1 M, (b) 0.2, (c) 0.2Se, (d) 0.2Te, and (e) Se_0.15_Te_0.05_ electrolytes before and after 100 cycles.

**References**

1. Zhao, M.; Li, X.-Y.; Chen, X.; Li, B.-Q.; Kaskel, S.; Zhang, Q.; Huang, J.-Q., *eScience* **2021,** *1* (1), 44-52.

2. Zhou, J.; Shu, C.; Zhang, Q.; Tang, W.; Wu, Y., *Science China Materials* **2025,** *68* (1), 207-216.

3. Ding, Y.; Li, X.; Chen, Y.; Pi, Y.; Yu, J.; Yuan, L.; Wang, F., *Chemical Engineering Journal* **2024,** *482*, 148803.

4. Zhang, W.; Ma, F.; Wu, Q.; Zeng, Z.; Zhong, W.; Cheng, S.; Chen, X.; Xie, J., *ENERGY & ENVIRONMENTAL MATERIALS* **2023,** *6* (3), e12369.

5. Sun, J.; Zhang, K.; Fu, Y.; Guo, W., *Nano Research* **2023,** *16* (3), 3814-3822.

6. Fan, Q.; Li, B.; Si, Y.; Fu, Y., *Chemical Communications* **2019,** *55* (53), 7655-7658.

1. Corresponding author. *E-mail address*: [haiweiwufly@163.com](mailto:haiweiwufly@163.com) (H Wu); [wuhaiwei@sust.edu.cn](mailto:wuhaiwei@sust.edu.cn) ; [zjli@sust.edu.cn](mailto:zjli@sust.edu.cn) (Z Li); [lin.zhang@fkp.uni-hannover.de](mailto:lin.zhang@fkp.uni-hannover.de) (L Zhang). [↑](#footnote-ref-1)
